# Beyond Blood Smears: Qualification of *Plasmodium* 18S rRNA as a Biomarker for Controlled Human Malaria Infections

**DOI:** 10.4269/ajtmh.19-0094

**Published:** 2019-04-22

**Authors:** Annette M. Seilie, Ming Chang, Amelia E. Hanron, Zachary P. Billman, Brad C. Stone, Kevin Zhou, Tayla M. Olsen, Glenda Daza, Jose Ortega, Kurtis R. Cruz, Nahum Smith, Sara A. Healy, Jillian Neal, Carolyn K. Wallis, Lisa Shelton, Tracie (VonGoedert) Mankowski, Sharon Wong-Madden, Sebastian A. Mikolajczak, Ashley M. Vaughan, Stefan H. I. Kappe, Matt Fishbaugher, Will Betz, Mark Kennedy, Jen C. C. Hume, Angela K. Talley, Stephen L. Hoffman, Sumana Chakravarty, B. Kim Lee Sim, Thomas L. Richie, Anna Wald, Christopher V. Plowe, Kirsten E. Lyke, Matthew Adams, Gary A. Fahle, Elliot P. Cowan, Patrick E. Duffy, James G. Kublin, Sean C. Murphy

**Affiliations:** 1 Department of Laboratory Medicine, Center for Emerging and Re-emerging Infectious Diseases, University of Washington, Seattle, Washington;; 2 Center for Global Infectious Disease Research, Seattle Children’s Research Institute (formerly the Center for Infectious Disease Research), Seattle, Washington;; 3 Division of Pediatric Infectious Diseases, Department of Pediatrics, University of Washington, Seattle, Washington;; 4 Laboratory of Malaria Immunology and Vaccinology, National Institute of Allergy and Infectious Diseases, National Institutes of Health, Bethesda, Maryland;; 5 Sanaria, Inc., Rockville, Maryland;; 6 Division of Allergy and Infectious Diseases, Department of Medicine, University of Washington, Seattle, Washington;; 7 Duke Global Health Institute, Duke University, Durham, North Carolina;; 8 Center for Vaccine Development and Global Health, University of Maryland School of Medicine, Baltimore, Maryland;; 9 Microbiology Service, Department of Laboratory Medicine, Clinical Center, National Institutes of Health, Bethesda, Maryland;; 10 Partners in Diagnostics, Rockville, Maryland;; 11 Vaccine and Infectious Diseases Division, Fred Hutchinson Cancer Research Center, Seattle, Washington;; 12 Seattle Malaria Clinical Trials Center, Fred Hutch Cancer Research Center, Seattle, Washington;; 13 Department of Microbiology, University of Washington, Seattle, Washington

## Abstract

18S rRNA is a biomarker that provides an alternative to thick blood smears in controlled human malaria infection (CHMI) trials. We reviewed data from CHMI trials at non-endemic sites that used blood smears and *Plasmodium* 18S rRNA/rDNA biomarker nucleic acid tests (NATs) for time to positivity. We validated a multiplex quantitative reverse transcription–polymerase chain reaction (qRT-PCR) for *Plasmodium* 18S rRNA, prospectively compared blood smears and qRT-PCR for three trials, and modeled treatment effects at different biomarker-defined parasite densities to assess the impact on infection detection, symptom reduction, and measured intervention efficacy. Literature review demonstrated accelerated NAT-based infection detection compared with blood smears (mean acceleration: 3.2–3.6 days). For prospectively tested trials, the validated *Plasmodium* 18S rRNA qRT-PCR positivity was earlier (7.6 days; 95% CI: 7.1–8.1 days) than blood smears (11.0 days; 95% CI: 10.3–11.8 days) and significantly preceded the onset of grade 2 malaria-related symptoms (12.2 days; 95% CI: 10.6–13.3 days). Discrepant analysis showed that the risk of a blood smear–positive, biomarker-negative result was negligible. Data modeling predicted that treatment triggered by specific biomarker-defined thresholds can differentiate complete, partial, and non-protective outcomes and eliminate many grade 2 and most grade 3 malaria-related symptoms post-CHMI. *Plasmodium* 18S rRNA is a sensitive and specific biomarker that can justifiably replace blood smears for infection detection in CHMI trials in non-endemic settings. This study led to biomarker qualification through the U.S. Food and Drug Administration for use in CHMI studies at non-endemic sites, which will facilitate biomarker use for the qualified context of use in drug and vaccine trials.

## INTRODUCTION

In 2016, *Plasmodium* parasites caused 219 million cases and 435,000 malaria-attributable deaths.^1^ The standard approach for the detection of these burdensome parasites has long been through microscopic examination of thick and thin Giemsa-stained blood smears, now often supplemented with rapid diagnostic tests. Thick blood smears (called “blood smears” hereafter) have also served as the gold standard for controlled human malaria infection (CHMI) studies.^2^ Controlled human malaria infection studies are clinical trials where experimental infection with sporozoite-stage parasites are conducted to test early stage vaccine and drug candidates. The blood smear limit of detection is ∼4–20 parasites/μL depending on the number of fields evaluated,^3,4^ which means that blood smears cannot usually detect parasites as they first emerge from the liver 6–7 days post-CHMI. Thus, by the time a *Plasmodium* infection becomes blood smear–positive post-CHMI in nonimmune persons, many participants are symptomatic. Over the past 20 years, sensitive nucleic acid tests (NATs) that afford earlier detection at lower parasite densities have been developed (reviewed in Refs. 5 and 6). Methods have included single-step and nested PCRs with electrophoresis gel–based detection, DNA dye- or probe-based real-time quantitative PCR (qPCR), nucleic acid–based sequence amplification (NASBA), and real-time quantitative reverse transcription–PCR (qRT-PCR) with probe-based detection.

The most common NAT targets of *Plasmodium* are its conserved 18S rRNA-coding genes (rDNA) by qPCR or the expressed 18S rRNAs themselves by qRT-PCR or NASBA. rRNA expression is relatively stage specific,^7–9^ with asexual (A)-type 18S rRNAs more highly expressed in erythrocyte-stage parasites and sexual (S)-type 18S rRNAs more abundant in mosquito stages.^8^ Each *Plasmodium* parasite expresses thousands of 18S rRNAs from a few coding genes,^10–12^ making it even possible to detect single parasites in a 0.05–1 mL blood sample by qRT-PCR or NASBA.^10,12,13^ Because of the difference in rRNA versus rDNA copy number per parasite, extractions for DNA qPCR generally require larger volumes of blood than those for qRT-PCR or NASBA to achieve the same parasite limit of detection. Initially, malaria NATs were used retrospectively in trials, but as the techniques matured, such methods have been increasingly used as primary endpoint assays in CHMI trials in non-endemic sites.^14–16^

Following CHMI, immunologically naive volunteers usually become blood smear positive 10–12 days later. However, in such persons, the blood stage begins approximately 6 days post-CHMI, as determined by in vitro culture of clinical samples^17,18^ and by NATs.^13^ Traditionally, CHMI participants were monitored by once- or twice-daily blood smears and received treatment on becoming patent. Dangerously, high-density or prolonged infections do not occur with this approach—to our knowledge, there have been no severe malaria cases or deaths in any CHMI trial to date. Whereas some CHMI participants at non-endemic sites are asymptomatic on blood smear patency,^13,19^ malaria-related symptoms (e.g., headache, myalgia, fever, chills, sweats, nausea, vomiting, and diarrhea) up to and including grade 3 symptoms often commence days earlier.^10,20–23^ Although well-controlled, sensitive assays for 18S rRNA/rDNA have been used in the context of CHMI trials, regulatory qualification of this biomarker^24^ has not been previously achieved. Here, analytical/clinical validation and regulatory qualification of the *Plasmodium* 18S rRNA/rDNA biomarker for non-endemic site CHMI studies are reported.

## MATERIALS AND METHODS

### Literature review.

Peer-reviewed publications of Pf CHMI that used *Plasmodium* 18S rRNA and/or rDNA assays and blood smears were identified in PubMed. Publicly available data (main manuscripts and Supplemental Information) were evaluated. Subject-level data were not evaluated unless publicly available.

### *Plasmodium* 18S rRNA qRT-PCR assay.

Extraction and amplification for the third-generation biomarker assay were entirely performed on the Abbott m2000sp and m2000rt instruments, respectively (Abbott Molecular, Niles, IL). Ethylenediamine tetraacetic acid (EDTA) venous whole blood was collected from trial participants, and 50 µL was mixed with 2 mL of NucliSENS lysis buffer (bioMérieux, Durham, NC). One milliliter of lysate was processed by the m2000sp using mSample RNA preparation kit (Abbott Molecular), and the m2000 whole blood RNA extraction protocol was followed by automated pipetting of mastermix and template into 96-well plates.

Our multiplex approach simultaneously evaluated a pan-*Plasmodium* 18S rRNA target, a *Plasmodium falciparum*–specific 18S rRNA target, and a human housekeeping mRNA target. The triplex qRT-PCR reaction was performed using 35 μL SensiFAST^™^ Probe Lo-ROX One-Step Kit (Bioline, Taunton, MA) and 15 μL of extracted eluate. Primers/probes were as follows for the pan-*Plasmodium* 18S rRNA segment (Forward PanDDT1043F19: 5′-AAAGTTAAGGGAGTGAAGA-3′; Reverse PanDDT1197R22: 5′-AAGACTTTGATTTCTCATAAGG-3′; Probe: 5′-[CAL Fluor Orange 560]-ACCGTCGTAATCTTAACCATAAACTA[T(Black Hole Quencher-1)]GCCGACTAG-3′[Spacer C3]), a Pf-specific 18S rRNA sequence (Forward PfDDT1451F21: 5′-GCGAGTACACTATATTCTTAT-3′; Reverse PfDDT1562R21: 5′-ATTATTAGTAGAACAGGGAAA-3′; Probe: 5′-[6-FAM]-ATTTATTCAGTAATCAAATTAGGAT-3′[Black Hole Quencher-1]), and the human TATA-Binding Protein (TBP) mRNA (Forward: 5′-GATAAGAGAGCCACGAACCAC-3′; Reverse: 5′-CAAGAACTTAGCTGGAAAACCC-3′; Probe: 5′-[Quasar 670]-CACAGGAGCCAAGAGTGAAGAACAGT-3′[Black Hole Quencher-2]). Probes were dual high performance liquid chromatography-purified (LCG BioSearch Technologies, Novato, CA). All probes and human TBP primers were at 0.1 µM final, Pf-specific primers 0.4 µM, and pan-*Plasmodium* primers 0.2 µM. Cycling conditions were reverse transcription (10 minutes) at 48°C, denaturation (2 minutes) at 95°C, and 45 PCR cycles of 95°C (5 seconds) and 50°C (35 seconds).

### Pf culture.

Control samples and some validation samples were generated from Pf 3D7 strain parasites cultured using the methods of Trager and Jensen as previously reported.^10^ ABO blood group-matched whole blood was used for diluting erythrocyte cultures to prevent rosetting.

### Calibration and reporting.

A custom lot of quantified Armored RNA encoding full-length Pf 18S rRNA (Asuragen, Austin, TX) was used as an absolute 18S rRNA calibrator by addition to lysed Pf-negative whole blood before extraction. Copies/mL of whole blood were converted to estimated parasites/mL of whole blood by dividing by the per-parasite copy number conversion factor (see Results). Quantitative results were reported for estimated parasite densities ≥ 20 parasites/mL, qualitative “low-positive” results for results equivalent to 10 to < 20 estimated parasites/mL, and “not detected” for lower and undetectable results.

### Quality assurance.

High (4 × 10^5^ parasites/mL), low (8 × 10^2^ parasites/mL), and negative controls were tested in each assay run and monitored using 30-run Levey-Jennings plots. Westgard 1_3s_ (run rejected if control > 3 SDs from expected value), 2_2s_ (run rejected if two consecutive controls are > 2 SDs from expected value), and 12_x_ rule (run rejected if 12 consecutive control measurements are on one side of the mean).^25^ Human TBP mRNA was monitored as an endogenous internal control. The laboratory exchanged samples with outside laboratories for external quality assurance (EQA) and enrolled in the WHO EQA scheme for malaria nucleic acid amplification testing (http://www.who.int/malaria/publications/atoz/NAAT-EQA-manual/en/).

### Discrepant analysis.

Discrepant analyses were performed following Food and Drug Administration (FDA) guidelines.^26^ Agreement and CIs were calculated following the Clinical and Laboratory Standards Institute (CLSI) standards.^27^ Tests used to assess the primary result (pan-*Plasmodium* channel of biomarker assay) included the Pf-specific channel of the current assay, the first generation of the University of Washington (UW) Pf 18S rRNA qRT-PCRs,^10^ and assays performed by outside laboratories, including the University of Maryland 18S rRNA biomarker qRT-PCR (University of Maryland),^28^ the Laboratory for Malaria Immunology and Vaccinology 18S rDNA qPCR (unpublished, J. Neal, personal communication), and the NIH Clinical Center 18S rDNA PCR (NIH).^29^

### Human clinical samples.

Leftover clinical whole blood specimens (50 μL) from local hospitals were used under a protocol approved by the UW Institutional Review Board (IRB) (Protocol 47026, S. Murphy). Samples were also obtained from the following completed IRB-approved clinical trials. The MC-001 Demo Trial (IND 14224; ClinicalTrials.gov NCT01058226) conducted at the Center for Infectious Disease Research (now Seattle Children’s Research Institute) was a single-center, open-label phase 1 trial to demonstrate the ability to conduct CHMI trials under an investigational new drug (IND) application and obtain immunological endpoints after one exposure.^10,21^ The MC-003 trial (IND 14752; ClinicalTrials.gov NCT01500980) was a phase 1, randomized, partial double-blind, placebo-controlled study at the same site by Infection-Treatment-Vaccination using chloroquine and primaquine. The PfSPZ-CVac pyrimethamine (PYR) study (NIAID Protocol Number 15-I-0169; IND 16650; ClinicalTrials.gov NCT03083847) was a phase 1 study conducted at the NIH Clinical Center of Sanaria^®^ PfSPZ-CVac (*P. falciparum* sporozoite-chemoprophylaxis vaccination^30^) using chloroquine and PYR as the prophylactic drugs. The CHMIs for the MC-001 and MC-003 studies were given by five infected mosquito bites per participant, whereas CHMI for the NIH PfSPZ-CVac PYR study was by direct venous injection (DVI) of 3.2 × 10^3^ aseptic, purified, cryopreserved Pf sporozoites (Sanaria PfSPZ Challenge).^20,31^ In all studies, participants provided informed consent; additional details are available in the Supplemental Information and at ClinicalTrials.gov. Studies were evaluated for onset of blood smear positivity, 18S rRNA positivity, and malaria-related symptom onset and severity. Solicited malaria-related adverse events (AEs) included temperature −≥ 38°C, fever, malaise, myalgia, headache, nausea, vomiting, chills, lower back pain, diarrhea, abdominal pain, arthralgia, and chest pain. Temperature grading was grade 1 (38.0–38.4°C), 2 (38.5–38.9°C), 3 (39.0–40°C), and 4 (> 40°C). Symptom grading used comparable scales as defined in individual trial protocols.

### Data management and statistics.

Clinical and laboratory data for included trials were managed by DF/Net Research (Seattle, WA). For most comparisons, paired or unpaired Student’s *t*-tests were used, depending on the nature of the data. Food and Drug Administration and CLSI guidance were followed for calculating CIs of assay data as indicated. Statistical significance was considered at *P* < 0.05.

## RESULTS

### Literature review: *Plasmodium* 18S rRNA/rDNA biomarker versus blood smears in CHMI trials at non-endemic sites.

**Figure 1.**
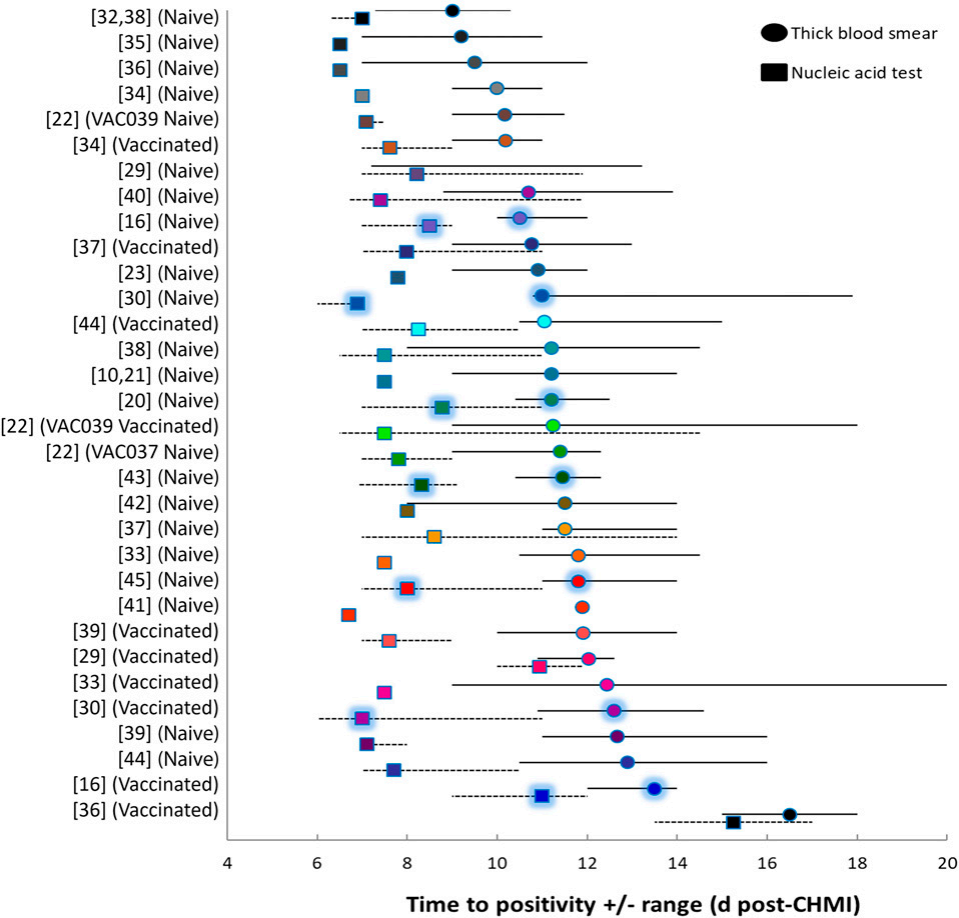
Time to positivity for *Plasmodium* 18S rRNA/rDNA biomarker and blood smears in published controlled human malaria infection (CHMI) studies. Day 0 is the day of CHMI. Squares indicate mean onset of biomarker detection and circles indicate blood smear positivity, unless otherwise denoted as median values in Supplemental Table 1. Shadowed data points are PfSPZ direct venous injection CHMI, and non-shadowed points are mosquito bite CHMI. Error bars indicate minimum/maximum ranges, if available. In some studies, mean or range data could not be determined from the primary publications. *Y*-axis labels indicate references in the main text, with naive or vaccinated subgroups from each study denoted in parentheses.

Twenty-two CHMI studies at non-endemic sites were reviewed on 488 volunteers (290 naïve, 198 vaccinated), where both blood smears and biomarker NATs were performed^10,16,20–23,29,30,32–45^ (Figure 1, Supplemental Table 1). Data on participants administered intramuscular or intradermal PfSPZ Challenge were not included. Controlled human malaria infection was by either three to seven infectious mosquito bites or DVI of 3.2 × 10^3^ PfSPZ Challenge (Sanaria, Rockville, MD). Across all studies and groups, the biomarker time to positivity (TTP) was shorter than that for blood smears (Figure 1), with biomarker assays becoming positive before blood smears (mean difference: 3.4 days; 95% CI: 2.8–4.0). The reported limit of detection for the assays used in the included studies ranged from 10 to 500 parasites/mL.

### Immunologically naive participants at non-endemic studies.

Naive participants at non-endemic sites included 248 individuals who underwent mosquito bite CHMI and 42 who underwent CHMI by DVI of PfSPZ Challenge (Supplemental Table 1). For mosquito bite CHMI, biomarker positivity (mean: 7.4 days; range: 6.3–14 days) occurred 3.6 days earlier (range: 2–5.2 days) than blood smears (mean: 10.9 days; range: 7–16 days) (Table 1). For the DVI CHMIs, biomarker positivity (mean: 8.0 days; range: 7–11 days) occurred 3.2 days earlier (range: 2–4.1 days) than blood smears (mean: 11.2 days; range: 10–17.9 days). Blood smear and biomarker TTPs were not significantly different for CHMI by mosquito bites versus PfSPZ DVI. Positive blood smears occurred at biomarker-estimated parasite densities ranging from 1 × 10^3^ to 5.4 × 10^5^ parasites/mL (1–540 parasites/μL). Exact timing of the onset of malaria-specific symptoms could not be determined from the primary publications.

**Table 1:** Time to positivity differences for naive and previously vaccinated participants in published CHMI studies at non-endemic sites

Group	Datasets (*n*)	Subjects (*n*)	TBS+ (%)	TBS TTP mean (range)	Biomarker TTP mean (range)	Δ_TBS-Biomarker_ (SD)	*P*-value
Immunologically naive volunteers							
3–7 mosquito bites	17	248	98.1	10.9 (7–16)	7.3 (6.7–14)	3.6 (1.1)	< 0.0001
3.2 × 10^3^ PfSPZ DVI	5	42	100	11.2 (10–17.9)	8.0 (6–11)	3.2 (0.7)	< 0.001
Previously vaccinated volunteers							
Efficacy > 50%*	3	48	29.2	13.7 (10.9–18)	11.1 (6–17)	2.7 (2.6)	0.21
Efficacy ≤ 50%†	7	150	92.7	11.6 (9–20)	8.2 (6.5–14.5)	3.4 (0.9)	< 0.0001

CHMI = controlled human malaria infection; DVI = direct venous injection; TBS = thick blood smear; TTP = time to positivity. Time to positivity and range data in days post-CHMI; *P*-values comparing TBS TTP with biomarker TTP using paired Student’s *t*-tests for study- or cohort-specific data.

* Includes two CHMIs by mosquito bites and one by DVI of PfSPZ Challenge.

† Includes six CHMIs by mosquito bites and one by DVI of PfSPZ Challenge.

### Previously vaccinated participants at non-endemic studies.

One hundred ninety-eight previously vaccinated individuals affected by CHMI through mosquito bites (*n* = 165) or DVI (*n* = 33) were evaluated (Supplemental Table 1). In some cases, volunteers participated in an initial CHMI and, if initially protected, in a later repeat CHMI; in such cases, each CHMI counted as an individual CHMI for our analyses. Previously vaccinated participants were stratified on the basis of whether the vaccine cohort was highly protected against CHMI or not. Three included groups showed efficacy ≥ 50% (“effective” group for this analysis),^29,30,36^ and those with efficacy < 50% were considered separately.^16,22,33,34,37,39,44^ For vaccines deemed effective for this analysis, 34/48 participants showed complete protection (i.e., biomarker-negative throughout). Among incompletely protected participants in the effective cohorts, biomarker assays were positive 2.7 days earlier (mean: 11.1 days; range: 6–17 days) than blood smears (mean: 13.7 days; range: 10.9–18 days) (Table 1). For vaccines with efficacy < 50%, biomarker positivity occurred (mean: 8.2 days; range: 6.5–14.5 days) 3.4 days earlier (range: 2.5–4.9 days) than blood smears (mean: 11.6 days; range: 9–20 days). Compared with naive controls, blood smears and 18S rRNA/rDNA biomarker TTPs for the effective vaccine group were significantly shorter than those from naive participants (*P* = 0.001 and *P* < 0.001, respectively), whereas there were no significant differences between the ineffective vaccine group and the naive controls for either endpoint.

### Analytical validation of the *Plasmodium* 18S rRNA multiplex qRT-PCR.

A multiplex *Plasmodium* 18S rRNA qRT-PCR was analytically validated in accordance with CLSI guidelines.^46^ Key characteristics and assay performance are summarized in the following paragraphs. The assay detects and quantifies pan-*Plasmodium* and Pf-specific regions of the 18S rRNA and a human TBP endogenous control mRNA. Pan-*Plasmodium* primers (PanDDT1043F19/PanDDT1197R22) and a probe are 100% conserved in all human-infecting plasmodia (Supplemental Figure 1, Supplemental Table 2); in rodent-infecting species *Plasmodium yoelii*, *Plasmodium chabaudi*, and *Plasmodium berghei*; and in primate-infecting species *Plasmodium cynomolgi* and *Plasmodium reichenowi*. The pan-*Plasmodium* amplicon contains nucleotide variation that permits species identification by amplicon sequencing. The Pf target used novel Pf-specific primers (PfDDT1451F21/PfDDT1562R21) and a published Pf probe^11^ that are 100% matched to A-type 18S rRNA genes MAL5_18S/PF3D7_0531600 and MAL7_18S/PF3D7_0725600 and to 10 full- or partial-length Pf A-type 18S rRNA sequences in GenBank (not shown). Pf reagents have mismatches to Pf S-type genes and are absent from non-Pf 18S rRNA genes (Supplemental Figure 2). These Pf primers also detected additional Pf infections in field studies that were missed by earlier generations^10,11^ of the assay, demonstrating improved coverage of variant Pf strains (S. Das, G. Domingo, S. Murphy, personal communication).

The method was tested for accuracy, precision, analytical sensitivity, sample stability and analytical specificity (interferences), reportable range, and carryover using whole blood samples. Briefly, standard curves demonstrated an efficient reaction and stable, reproducible performance (pan-*Plasmodium* slope −3.38 cycles/log_10_ copies/mL lysate, 95% CI: −3.17 to −3.59; intercept 41.9 cycles; 95% CI: 39.4–44.3 cycles; *r*^2^: 0.998). The Armored RNA calibrator was used to generate a conversion factor for 18S rRNA copies per ring-stage parasite of 7.4 × 10^3^ 18S rRNA copies/parasite (mean and median; 95% CI: 6.17 × 10^3^ to 8.71 × 10^3^; *n* = 22 samples) following our previously published approach^10,11^ using EQA samples extensively characterized by qPCR and qRT-PCR^47^; the conversion factor was within 2-fold of the values determined for the earlier generations of the assay.^10,11^

Among 106 samples of known density (range: 1 × 10^2^ to 4 × 10^5^ parasites/mL), the average log_10_ difference (bias) between measured and expected results was +0.11 log_10_ parasites/mL (95% CI: −0.23 to 0.44 log_10_ parasites/mL) in the pan-*Plasmodium* channel, with no evidence of concentration-dependent differences in recovery (Supplemental Figure 3). Within-run and between-run precision^48^ were acceptable (Supplemental Table 3). For low-density samples, the pan-*Plasmodium* channel detected 100% (10/10), 95% (19/20), and 95% (19/20) of samples at nominal concentrations of 100, 50, and 20 parasites/mL, respectively (Supplemental Table 4). To further evaluate assay sensitivity, Armored RNA was added to whole blood samples to create samples equivalent to low parasite densities. Positive Armored RNA results included 21/21 samples at a copy number equivalent to one parasite per 50 μL of whole blood (1.48 × 10^5^ copies/mL of blood or 20 parasites/mL) and 20/20 at a copy number equivalent to one-third of a parasite per 50 μL of whole blood (5.3 × 10^4^ copies/mL of blood or ∼7 parasites/mL). The limit of quantification determined by CLSI methods^49^ was 20 parasites/mL using Armored RNA added to lysed malaria-negative whole blood (1.48 × 10^5^ copies/mL whole blood) (Supplemental Table 5), for a reportable range of 20 to 1 × 10^7^ parasites/mL. No carryover was noted (44 known negative samples processed immediately after paired high positives). The assay detects asexual-stage *Plasmodium* parasites as well as gametocytes and sporozoites. There were no interferences from hemolysis (to 10 times upper limit of normal [ULN] for free hemoglobin), lipemia (to 6.5-fold ULN), bilirubinemia (to 8-fold ULN), heparin (to 40 U.S. Pharmacopeia heparin units/mL), *Candida albicans* (to 4 × 10^3^ cfu/mL), *Cytomegalovirus* (5 × 10^4^ IU/mL), Epstein–Barr virus (5 × 10^4^ IU/mL), HIV-1 (viral load 4.76 log_10_ RNA copies/mL), HIV-2 (viral load 3.24 log_10_ RNA copies/mL), or *Trypanosoma brucei* spp. (1 × 10^6^ parasites/mL). Leukocytosis up to 25 × 10^9^ cells/L did not interfere, but more severe leukocytosis led to biomarker underestimation (−0.34 log_10_ estimated parasites/mL less than controls). Weak cross-reactivity for high-density *Babesia microti* samples (5% parasitemia) occurred in the pan-*Plasmodium* channel (not the Pf channel); a *Babesia*-specific assay is available to discriminate.^50^

Stability studies showed that the *Plasmodium* 18S rRNA is stable in parasite-containing EDTA whole blood samples (2 × 10^2^ estimated parasites/mL) for 96 hours at room temperature or 72 hours at 4°C before lysis buffer addition (Supplemental Table 6). Whole blood could also be frozen and thawed one time with subsequent processing into lysis buffer on thawing without significant biomarker degradation (*P* = 0.21, *n* = 9 paired samples), whereas repeated freeze-thawing resulted in more significant degradation (*P* = 0.003, *n* = 9 paired samples) (Supplemental Figure 4). Finally, correlation studies showed close agreement with earlier assay generations^10,11^ (first- and third-generation assays: *n* = 68 samples, slope: 1.06, intercept: 0.03 log_10_ parasites/mL, *r*^2^: 0.91; second- and third-generation assays: *n* = 98 samples, slope: 1.00, intercept: 0.10 log_10_ parasites/mL, *r*^2^: 0.99).

### Clinical validation of the 18S rRNA biomarker.

We performed a clinical validation of the *Plasmodium* 18S rRNA to support biomarker qualification through the FDA.^51^ We tested samples from three CHMI studies from non-endemic sites (MC-001, MC-003, and PfSPZ-CVac PYR; Table 2), in which blood smears and second- or third-generation *Plasmodium* 18S rRNA qRT-PCR were performed. Quantitative reverse transcription–PCR and blood smear results (one sample per day for each protocol-defined testing day) and malaria-related clinical data were included. Discrepant analyses were performed for two trials. Data for each study are described and evaluated in Supplemental Information and Supplemental Tables 7–11. Summary data are below.

**Table 2:** Studies included in the clinical validation

Study	No. at CHMI	CHMI route	No. TBS+	No. biomarker+	No. treated for infection
MC-001	6	5 bites	6	6	6
MC-003*	29	5 bites	26*	25	26*
PfSPZ-CVac PYR	21	3200 DVI	6	15	15

CHMI = controlled human malaria infection; DVI = direct venous injection; PYR = pyrimethamine; TBS = thick blood smear.

* One MC-003 participant was treated on the basis of a mislabeled blood smear specimen and was excluded from this analysis as described in the text. The analysis herein includes *n* = 28 participants, including 25 who were blood smear–positive and 18S rRNA biomarker positive.

### Combined analysis of *Plasmodium* 18S rRNA infection detection across all studies.

**Figure 2.**
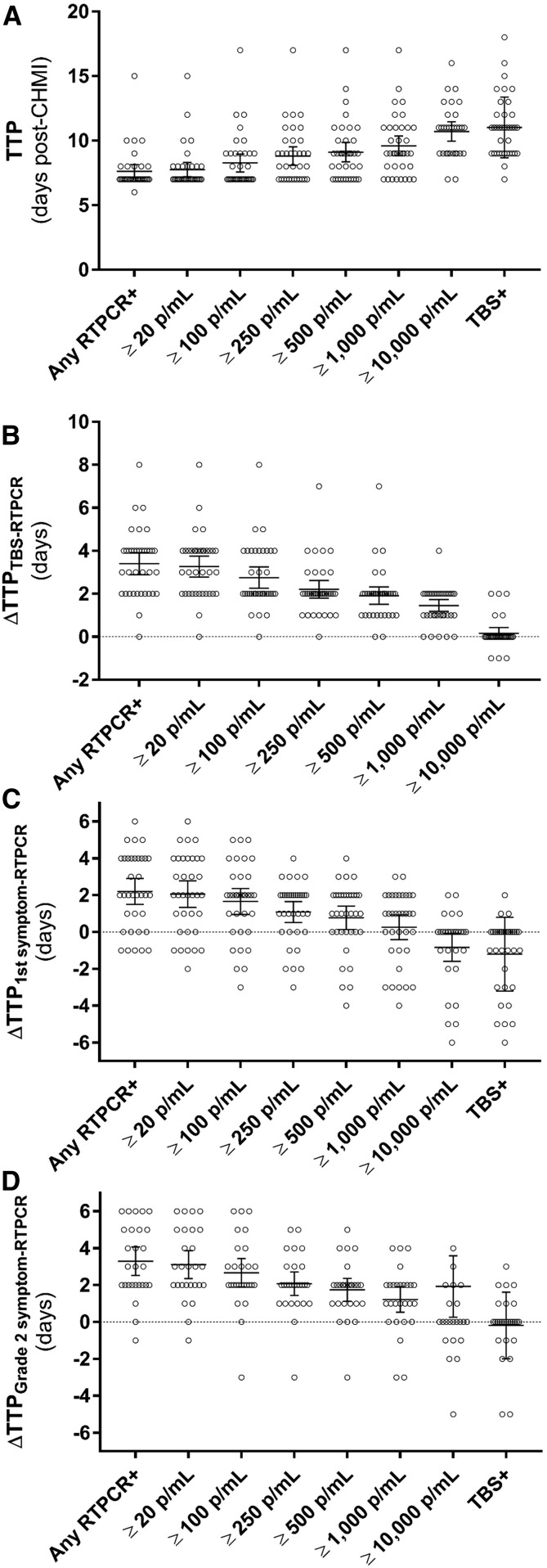
Differences in time to positivity (TTP) for *Plasmodium* 18S rRNA biomarker vs. blood smears or symptoms across all studies. (**A**) Time to positivity for quantitative reverse transcription PCR (qRT-PCR) biomarker-estimated parasite densities or to TBS positivity as shown. Differences in TTP for qRT-PCR biomarker compared with blood smears (**B**) using various biomarker-defined thresholds (any positive or quantitative positive ≥ 20 estimated parasites/mL to ≥ 10,000 estimated parasites/mL as shown). Differences in TTP for qRT-PCR biomarker compared with the first solicited malaria-related symptom of any grade (**C**) or first solicited grade 2 malaria-related symptom (**D**) using biomarker-defined thresholds as in **A**. Each data point corresponds to an individual participant. TBS = thick blood smear; p/mL = estimated parasites/mL. Error bars, 95% CI.

In the included trials, blood smear–positive participants showed the presence of the biomarker in peripheral blood *on* or *before* the time of blood smear positivity (*n* = 37). Across all such volunteers, the mean TTP for any level of biomarker positivity (including low positives) was 7.6 days (range: 6–15 days; *n* = 37), with correspondingly longer times needed to reach higher qRT-PCR–estimated densities (Table 3 and Figure 2A). By contrast, for blood smear–positive participants, mean blood smear TTP was 11.0 days (range: 7–18 days; *n* = 37). On average, biomarker positivity began 3.4 days (95% CI: 3.0–3.8 days) earlier than blood smear positivity. When modeled against different qRT-PCR–determined parasite densities, there were density-dependent accelerations compared with blood smears (Figure 2B, Table 4). For instance, the mean TTP difference between biomarker equivalent to ≥ 250 est. parasites/mL and blood smears was 2.2 days (95% CI: 1.9–2.6 days), whereas the difference was greater if a more sensitive biomarker-based threshold of 20 parasites/mL was used (3.3 days; 95% CI: 2.9–3.7 days).

**Table 3:** Onset of blood smear and *Plasmodium* 18S rRNA positivity and treatment for blood smear–positive participants

Days from CHMI to	Statistic	MC-001 (*N* = 6)	MC-003 infectivity and drug controls (*N* = 11)	MC-003 vaccinated (*N* = 14)	PfSPZ-Cvac PYR (*N* = 6)	All studies (*N* = 37)
Biomarker positive including low positives of ≥ 10 est. parasites/mL	Mean	7.7	6.9	7.5	9.2	7.6
	95% CI	6.4–8.9	6.7–7.1	6.9–8.1	6.1–12.2	7.1–8.1
Biomarker ≥ 20 est. parasites/mL	Mean	7.7	7.0	7.7	9.3	7.8
	95% CI	6.4–8.9	7.0–7.0	6.9–8.6	6.4–12.3	7.2–8.3
Biomarker ≥ 100 est. parasites/mL	Mean	8.2	7.0	8.2	10.8	8.3
	95% CI	6.8–9.6	7.0–7.0	7.3–9.1	7.2–14.4	7.6–9.0
Biomarker ≥ 250 est. parasites/mL	Mean	9.0	7.3	8.7	11.7	8.8
	95% CI	7.3–10.8	6.8–7.7	7.9–9.6	8.6–14.7	8.1–9.5
Biomarker ≥ 500 est. parasites/mL	Mean	9.3	7.5	9.0	12.2	9.1
	95% CI	7.4–11.3	6.9–8.0	8.0–10.0	9.7–14.7	8.4–9.9
Biomarker ≥ 1,000 est. parasites/mL	Mean	9.8	7.8	9.8	12.2	9.6
	95% CI	8.0–11.6	7.1–8.5	8.6–11.0	9.7–14.7	8.8–10.3
Biomarker ≥ 10,000 est. parasites/mL	Mean	11.2*	9.0*	11.0*	12.6*	10.7*
	95% CI	9.0–13.4	7.9–10.1	9.7–12.7	11.2–14.0	10.0–11.5
Positive blood smear	Mean	11.2	9.2	11.4	13.5	11.0
	95% CI	9.5–12.9	8.4–10.0	10.0–12.7	11.0–16.0	10.3–11.8
Treatment	Mean	11.2	9.2	11.4	13.3	11.0
	95% CI	9.5–12.9	8.4–10.0	10.0–12.7	10.8–15.9	10.2–11.8

CHMI = controlled human malaria infection; PYR = pyrimethamine.

* Fewer than the total number of participants reached a biomarker-calculated density of 10,000 estimated parasites/mL before treatment (MC-001”, *n* = 5; MC-003 infectivity/drug controls, *n* = 9; MC-003 vaccinated, *n* = 12; PfSPZ-Cvac PYR, *n* = 5; total *n* = 31).

**Table 4:** Comparisons of *Plasmodium* 18S rRNA versus blood smears and symptoms for all studies

Biomarker threshold (est. parasites/mL)	Mean days from biomarker to TBS positivity*			Mean days from biomarker to any related symptom†			Mean days from biomarker to any grade 2 related symptom‡		
	Mean difference	95% CI§	*P*-value	Mean difference	95% CI§	*P*-value	Mean difference	95% CI§	*P*-value
Any (+) (incl. low positives)	3.4	(3.0–3.8)	****	2.2	(1.6–2.7)	****	3.3	(2.7–4.0)	****
≥ 20	3.3	(2.9–3.7)	****	2.0	(1.5–2.6)	****	3.2	(2.6–3.8)	****
≥ 100	2.8	(2.4–3.2)	****	1.6	(1.0–2.1)	****	2.7	(2.1–3.3)	****
≥ 250	2.2	(1.9–2.6)	****	1.0	(0.5–1.4)	***	2.1	(1.6–2.6)	****
≥ 500	1.9	(1.6–2.3)	****	0.7	(0.2–1.2)	*	1.8	(1.3–2.2)	****
≥ 1,000	1.5	(1.2–1.7)	****	0.2	(−0.3 to 0.7)	0.25	1.2	(0.7–1.8)	***
≥ 10,000	0.2	(−0.1 to 0.4)	0.12	−0.9	(−1.5 to −0.3)	0.99	0.1	(−0.5 to 0.8)	0.37
Positive TBS	NA	NA	NA	−1.1	(−1.7 to −0.6)	0.99	−0.2	(−0.8 to 0.4)	0.70

NA = not applicable; TBS = thick blood smears. * *P* < 0.05; *** *P* < 0.001; **** *P* < 0.0001. *T*-tests performed among participants who eventually tested positive by TBS.

* H_O_: μ_TBS - BIOMARKER_ ≤ 0, H_A_: μ_TBS - BIOMARKER_ > 0.

† H_O_: μ_ANY SYMPTOM - BIOMARKER_ ≤ 0, H_A_: μ_ANY SYMPTOM - BIOMARKER_ > 0.

‡ H_O_: μ_GRADE 2 SYMPTOM - BIOMARKER_ ≤ 0, H_A_: μ_GRADE 2 SYMPTOM - BIOMARKER_ > 0.

§ One-sided CI.

For the included studies, the mean time for first malaria-related symptom of any grade was 9.7 days (range: 6–14 days; *n* = 37) and for first grade 2 malaria-related symptom was 12.2 days (range: 6–15 days; *n* = 28) (Table 5). The mean difference between any biomarker positivity and malaria-related symptom onset was 2.2 days (95% CI: 1.6–2.7 days). As for blood smears, modeling of different treatment thresholds indicates that treatment at lower densities is likely to mitigate symptom onset seen at higher densities up to and including blood smears (Figure 2C–D, Table 4).

**Table 5:** Summary of post-CHMI malaria-related symptoms in blood smear–positive participants

Days from CHMI to	Statistic	MC-001 (*N* = 6)	MC-003 infectivity and drug controls (*N* = 11)	MC-003 vaccinated (*N* = 14)	PfSPZ-Cvac PYR (*N* = 6)	All studies (*N* = 37)
First symptom (any grade)	*N*	6	11	14	4	35
	Mean (days)	9.7	8.2	9.8	12.8	9.7
	95% CI (days)	6.4–12.2	7.2–9.1	8.5–11.1	10.4–14.2	8.9–10.4
First grade 2 symptom	*N*	5	10	9	4	28
	Mean (days)	12.2	8.8	10.0	13.8	10.7
	95% CI (days)	10.6–13.3	7.9–9.6	8.2–11.8	12.2–14.7	9.7–11.6

CHMI = controlled human malaria infection; PYR = pyrimethamine.

The mean parasite density on becoming qRT-PCR positive was 950 estimated parasites/mL (range: 19–10,904; *n* = 37 by qRT-PCR) (Supplemental Table 12). At blood smear positivity, the mean parasite density by blood smear was 19,668 estimated parasites/mL (range: 1,700–82,000; *n* = 37) and 41,979 estimated parasites/mL by qRT-PCR (range: 1,687–162,152; *n* = 37) (Supplemental Table 12). Quantification by blood smears and qRT-PCR was moderately correlated (*r*^2^: 0.45; *n* = 37 samples), with qRT-PCR estimates slightly higher than those by blood smears (bias +0.28 log_10_ parasites/mL, 95% CI: −0.53 to +1.10 log_10_ parasites/mL; data not shown).

### Discrepant analyses support accuracy of prepatent detection of *Plasmodium* 18S rRNA.

*Plasmodium* 18S rRNA/rDNA biomarker assays have lower limits of detection and are therefore more analytically sensitive than blood smears. Thus, a common result in low-density infections is “biomarker positive, blood smear negative.” Volunteers in these studies who were initially qRT-PCR positive progressed to higher density infections and eventually became blood smear positive. To further assess the ability to reliably detect biomarker at sub-patent densities, all positive and negative samples from two of the studies (MC-001 and PfSPZ-CVac PYR) were subjected to discrepant analyses using published assays conducted in our laboratory and at collaborating centers (Supplemental Figure 5). There was 100% positive percent agreement between blood smears and the described pan-*Plasmodium* 18S rRNA qRT-PCR in both studies (Supplemental Table 13). As expected, negative percent agreement (NPA) between blood smears and pan-*Plasmodium* qRT-PCR was < 100% in all cases. As the limit of detection of comparator molecular assays approached that of the pan-*Plasmodium* 18S rRNA qRT-PCR, NPA between the pan-*Plasmodium* qRT-PCR result and the comparator test approached 100%. Discrepant analyses showed that the risk of a blood smear–positive/qRT-PCR–negative result is close to zero. These data show that qRT-PCR reliably detects *Plasmodium* 18S rRNA at submicroscopic densities.

### Evaluation of biomarker-defined treatment thresholds for CHMI studies.

Clinical trials with NAT endpoints can be designed to initiate treatment based on qualitative NAT positivity or on the basis of a quantitative NAT-defined threshold corresponding to a specific copy number or estimated parasite density. Waiting longer to treat post-CHMI may result in more symptoms, but allow better resolution of partially protected phenotypes between groups. To evaluate such trade-offs, correlations between biomarker, blood smears, and symptoms were evaluated for the three included trials. As described, lower biomarker levels were associated with progressively earlier infection detection compared with onset of blood smear patency and symptom onset (Table 4). The greatest acceleration was achieved using the most sensitive cutoff at the 20 parasite/mL assay limit of detection—as described earlier, blood smears and symptoms would be expected to trail 2–3 days behind this level of biomarker positivity. Of all symptoms that occur in CHMI studies, it is especially desirable to limit the frequency of grade 3 symptoms. Initiation of treatment based on the most sensitive biomarker threshold would be expected to eliminate nearly all grade 3 symptoms (Table 6).

**Table 6:** Predicted elimination of grade 3 AEs for biomarker-based thresholds

Grade 3 AEs	MC-001 (*N* = 6)	MC-003 infectivity and drug controls (*N* = 11)	MC-003 vaccinated (*N* = 14)	PfSPZ-Cvac PYR (*N* = 6)	All studies (*N* = 37)
As observed in the active studies					
Volunteers (%)	1 (16.7%)	5 (45.5%)	6 (42.9%)	3 (50.0%)	15 (40.5%)
Total number	4	14	16	4	38
Before or on the day of TBS positivity					
Volunteers (%)	1 (16.7%)	5 (45.5%)	5 (35.7%)	2 (33.3%)	13 (35.1%)
Total number	3	10	14	2	29
Before or on the day of biomarker equivalent to ≥ 250 estimated parasites/mL					
Volunteers (%)	1 (16.7%)	2 (9.1%)	1 (7.1%)	0 (0.0%)	4 (10.8%)
Total number	2	2	1	0	5
Before or on the day of any biomarker positivity					
Volunteers (%)	1 (16.7%)	1 (9.1%)	0 (0.0%)	0 (0.0%)	2 (5.4%)
Total number	2	1	0	0	3

AE = adverse event; PYR = pyrimethamine; TBS = thick blood smear. Adverse events were identified here as any AE that eventually became a grade 3 AE. For example, if a participant had a grade 1 AE on day 6, but it became a grade 3 on day 8, the starting date was counted as day 6.

Because not all assays achieve a limit of detection of 20 parasites/mL and because sampling bias may affect the limit of detection at such low densities, we also evaluated a cutoff of 250 estimated parasites/mL. This cutoff accelerated infection detection by > 2 days compared with blood smears and preceded malaria-related symptoms (any grade by 1.0 day and grade 2 by 2.1 days) (Table 4). The threshold of 250 parasites/mL would also be expected to precede nearly all grade 3 symptoms (Table 6).

**Figure 3.**
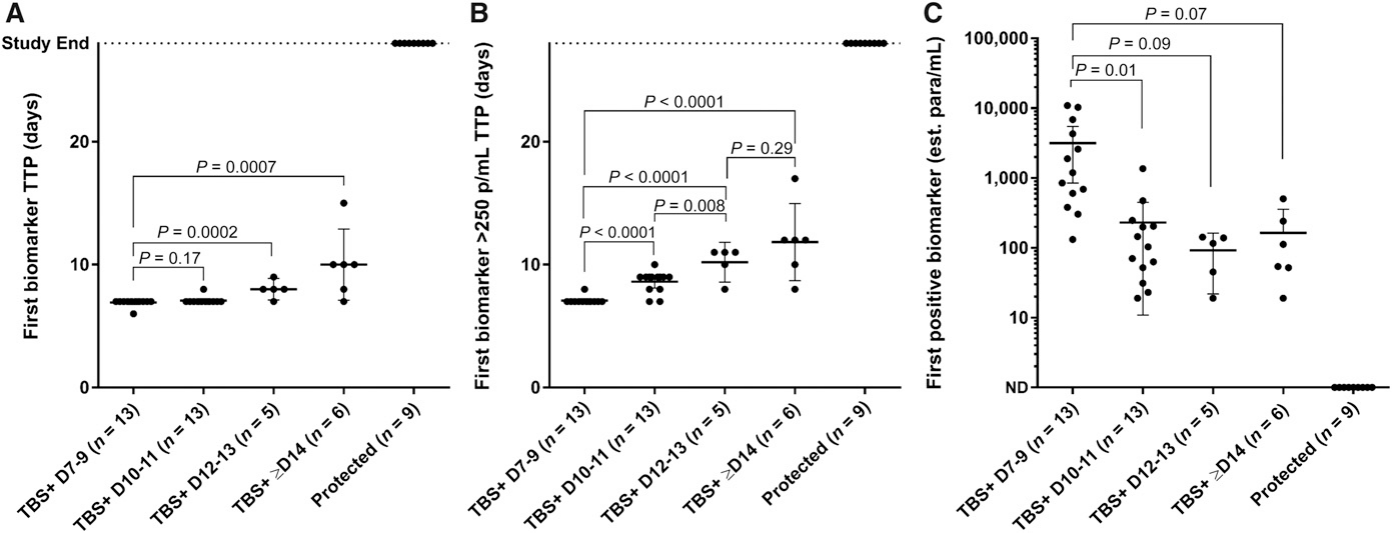
Ability of biomarker-based measurements to predict delayed blood smear patency. (**A**) Comparison of blood smear patency (participants grouped based on patency on days 7–9, 10–11, 12–13, or ≥ 14 days post-controlled human malaria infection) vs. first positive quantitative reverse transcription PCR–defined biomarker result of any density including qualitative low positives. (**B**) Comparison of blood smear patency as in **A** vs. time to first biomarker result equivalent to ≥ 250 estimated parasites/mL. (**C**) Comparison of blood smear patency as in **A** vs. estimated parasite density based on the first positive biomarker result. Each data point corresponds to an individual participant. TBS = thick blood smear; p/mL = estimated parasites/mL. *P*-values shown for two-sided unpaired Student’s *t*-tests. Error bars, 95% CI.

To evaluate the ability of different biomarker levels to differentiate degrees of partial protection, we also evaluated biomarker time to first positive, time to 250 estimated parasites/mL, and estimated parasite density at first positive against CHMI results stratified by day of blood smear patency. Subject-level data were binned into groups of no delay (blood smear–positive days 7–9, *n* = 13 participants), low delay (blood smear–positive days 10–11; *n* = 13 participants), moderate delay (blood smear–positive days 12–13; *n* = 5 participants), large delay (blood smear–positive day ≥ 14; *n* = 6 participants), or complete protection (*n* = 9 participants). Although time to first positive biomarker result could significantly differentiate the group with patency on days 7–9 from those on days 12–13 or ≥ 14, it could not do so for the day 10–11 group (Figure 3A). By contrast, time to ≥ 250 estimated parasites/mL by qRT-PCR closely mirrored the blood smear groupings and differentiated between most groups (Figure 3B). Early positive blood smears (days 7–9) were also accompanied by higher biomarker-based parasite densities than participants patent on day 10 or later (Figure 3C). Thus, these data and those of others^52^ support a *Plasmodium* 18S rRNA–based treatment threshold–based approach for differentiating outcomes of complete, partial, and zero protective efficacy.

## DISCUSSION

Controlled human malaria infection trials at non-endemic sites are increasingly used to test malaria drug and vaccine candidates and assess innate and naturally acquired resistance to malaria.^19,53^ In studies conducted at non-endemic sites using blood smear endpoints, many volunteers who develop parasitemia experience malaria-related AEs by the time parasites are microscopically detected. In these studies, delayed blood smear patency is used as an indicator of partial protection. Here, we provide analytical and clinical validation that, compared with blood smears, show that the *Plasmodium* 18S rRNA/rDNA biomarker can accelerate infection detection and reduce AEs while preserving the ability to discern complete, partial, and lack of protection post-CHMI. Review of the literature demonstrated consistently accelerated infection detection for the biomarker compared with blood smears in 22 published CHMI studies. Testing of archival samples from three CHMI trials using the analytically validated biomarker assay further confirmed these trends and showed that the biomarker is consistently detected approximately 7 days post-CHMI in immunologically naive persons, which is ∼ 2–4 days earlier than blood smear patency and symptom onset. The use of biomarker testing as the primary treatment endpoint is notably expected to eliminate most grade 3 symptoms and grade 3 AEs in CHMI studies at non-endemic sites. With an appropriately sensitive biomarker assay, discrepant analyses showed that the risk of a blood smear–positive/biomarker-negative result was close to zero. Thus, these data support the use of the *Plasmodium* 18S rRNA/rDNA biomarker as an alternative for blood smears in CHMI studies at non-endemic study sites. Not surprisingly, several recently published studies have used this biomarker as the primary endpoint for some or all drug and vaccine CHMI cohorts.^14–16^

These data were submitted to the FDA through the Center for Drug Evaluation and Research (CDER) Drug Development Tool Biomarker Qualification program^54,55^ to be considered for qualified use in non-endemic CHMI trials. Through a 2014–2018 process, the FDA recently qualified the biomarker^51^ for the following agency-wide context of use (COU): *Plasmodium* 18S rRNA/rDNA biomarker (reported in copies/mL blood) can be tested by a NAT for monitoring to inform initiation of treatment ≥ 6 days post-CHMI in *P. falciparum* studies at non-endemic sites. Notably, CHMI trial participants need to be biomarker negative at the start of a study and meet general CHMI inclusion/exclusion criteria. Potential interferences from extreme levels of leukocytosis and *Babesia* (as noted previously) were also noted by the FDA. These interferences are unlikely to be problematic in CHMI studies—individuals with leukocytosis > 25 × 10^9^/L would not be eligible for most studies at screening and *Babesia* cross-reactivity would only occur with high-density, clinically apparent infections. Such participants would be disqualified at screening by symptoms and by any reactive biomarker testing performed pre-CHMI. The Pf channel described in this multiplex assay does not cross-react with *Babesia*, and true Pf-positive results are expected to be pan- and Pf-channel positive. The FDA requests that biomarker data be reported in copies/mL—as such, we issue results in copies/mL as well as in estimated parasites/mL using a Pf-specific conversion factor. The FDA’s qualified COU considers any positive cycle threshold to be a qualitative positive biomarker result, but directs trial protocols to specify a biomarker threshold where treatment would be initiated—this threshold could be at the limit of detection or higher. Our study assessed a variety of thresholds and found that a density equivalent to 250 estimated parasites/mL is likely to reduce symptoms and allow differentiation between complete, partial, and lack of protection, which could be obscured if a much lower threshold was used. Another study found that a comparable threshold of 100 estimated parasites/mL behaved similarly.^52^ The FDA recommends that the WHO 18S rRNA calibrators be developed and that laboratories participate in EQA. Additional details about the COU and FDA-specific comments are available online.^51^ As described by the FDA, biomarker qualification status means that the biomarker can be used by drug and vaccine developers for the qualified COU in IND submissions, new drug applications, and biologics license applications without biomarker data resubmission or rereview by FDA. Additional study and future regulatory review are required to establish a qualified COU for other settings, such as endemic site CHMI and/or field studies.

This study has several limitations. First, this study did not address posttreatment clearance of the 18S rRNA biomarker, although these data are being compiled. Sensitive NATs can sometimes lead to prolonged positivity following adequate treatment. Second, human samples were limited to non-endemic CHMI trials reliant on NF54 strain Pf parasites. Other CHMI strains such as 7G8, NF135.C10, and NF166.C8 are in earlier stages of development and use,^14,56–59^ and biomarker agreement with NF54 and/or 3D7-based studies may need to be evaluated because prepatent periods can differ.^60^ Biomarker use in endemic sites will likely add other complexities such as preexisting infections, parasite strain and species variation, and higher rates of partial immunity. Third, this report describes studies where treatment was initiated by blood smear positivity and TTP modeling was based on sample collection times. If biomarker-positive participants are not treated until the day after becoming treatment eligible, the projected reduction in symptoms may not be fully realized. However, we and others have since conducted numerous clinical trials using the biomarker to initiate treatment including the DSM265 drug trial,^15^ published PfSPZ vaccine trials,^14,16^ an as-yet-unpublished multi-cohort drug trial (J. Kublin/S. Murphy, personal communication), an unpublished Sanaria PfSPZ-Cvac trial, and an ongoing study of genetically attenuated sporozoites administered by mosquito bites (L. Jackson, personal communication). In these studies, the biomarker has led to elimination of the domiciled hotel phase in the study designs. When these trials are published, the collective experience is likely to demonstrate accelerated infection detection and reduction in symptoms. Thus, the biomarker-based approach can safely accelerate infection detection in CHMI studies and provide nuanced protection data for evaluating early stage drugs and vaccines.

## Supplementary Files

Supplemental information, tables, and figures
