# Supplementary material for: Beyond Blood Smears: Qualification of Plasmodium 18S rRNA as a Biomarker for Controlled Human Malaria Infections
Source: Am J Trop Med Hyg. 2019 Apr 22;100(6):1466–76. doi: 10.4269/ajtmh.19-0094 (PMC6553913; doi:10.4269/ajtmh.19-0094)
Supplement: Supplementary file 1 [file tpmd190094.SD1.pdf]

The following are supplemental materials and will be published online only

SUPPLEMENTAL INFORMATION

Contents

SUPPLEMENTAL TEXT 2

MC-001 clinical study 2

MC-003 clinical study 2

NIH PfSPZ-CVac PYR study 3

SUPPLEMENTAL FIGURES 5

Figure S1. Mapping Pan-*Plasmodium* and *P. falciparum* specific primers/probes to consensus sequences of asexual-type 18S rRNAs of major *Plasmodium* species 5

Figure S2. Mapping Pan-*Plasmodium* and *P. falciparum* specific primers/probes to *P. falciparum* sexual-and asexual-type 18S rRNAs sequences 6

Figure S3. Difference plots for *P. falciparum* (A) and pan-*Plasmodium* (B) qRT-PCR 7

Figure S4. Stability of *Plasmodium* 18S rRNA in whole blood freeze-thaw experiments 8

Figure S5. Assays used for discrepant analyses 9

SUPPLEMENTAL TABLES 10

Table S1. Data summary of TBS and 18S rRNA/rDNA NATs in published CHMI trials 10

Table S2. Pan-*Plasmodium* assay primer/probe conservation to available GenBank sequences 12

Table S3. Precision analysis 13

Table S4. Analytical sensitivity analysis 14

Table S5. LoQ analysis at a nominal density of 20 parasites/mL 15

Table S6. One-sided unpaired *t*-tests of pre-lysis stability 16

Table S7. MC-001 study results 17

Table S8. MC-003 study results – infectivity/drug controls only (Arms 4 and 5) 18

Table S9. MC-003 study results – vaccinated participants only 19

Table S10. NIH PfSPZ-Cvac PYR study results - TBS positive volunteers only 20

Table S11. NIH PfSPZ-Cvac PYR study results – all treated volunteers 21

Table S12. Summary of parasite densities at first positive biomarker across all studies 22

Table S13. Agreement analysis 2x2 table for UW third generation Pan-*Plasmodium* 18S rRNA qRT-PCR method compared to reference and non-reference methods 23

SUPPLEMENTAL TEXT

MC-001 clinical study

MC-001 was a single site, open-label Phase 1 trial to demonstrate five mosquito bite CHMI under an IND (Ref. 21). Six healthy adult subjects were enrolled and developed malaria-related signs and symptoms. All became 18S rRNA positive [average 7.7 days (95%CI 6.4-8.9 days)] and later thick blood smear (TBS) positive [average 11.2 days (95%CI 9.5-12.8 days)] for a 3.5 day difference (**Table S7**). On average, the first malaria-related symptom (any grade) occurred at 9.7 days (95%CI 6.4-13.0 days) and Grade 2 malaria-related symptoms at 12.2 days (95%CI 10.6-13.8 days). RT-PCR-estimated parasite densities were similar to TBS-determined densities on the day of patency. Using a modeled treatment threshold of ≥250 estimated parasites/mL, RT-PCR achieved accelerated infection detection by 2.2 days (95%CI 1.7-2.6 days) versus TBS and 0.7 days (95%CI: -1.4 to 2.7 days) versus malaria-related symptoms. Since subjects were immunologically-naïve, this study did not test for differences in protection by TBS versus biomarker.

MC-003 clinical study

The MC-003 study was a single center, randomized, partially double-blinded, placebo-controlled Phase 1 trial of Infection-Treatment-Vaccination (ITV) using CQ with or without PQ in healthy adult subjects. Vaccinations were followed by five mosquito bite CHMI in all eligible vaccinated and infectivity control subjects. Data was included from CHMI only. Protocol-defined study arms were as follows (total number of subjects that completed CHMI):

- Pilot Arm 1a: PQ/CQ ITV three times starting PQ two days post-vaccination (n=2)
- Pilot Arm 1b: PQ/CQ ITV three times starting PQ three days post-vaccination (n=2)
- Arm 2: PQ/CQ ITV three times starting PQ one day post-vaccination (n=11)
- Arm 3: CQ-only ITV three times (n=3)
- Arm 4: Drug control treatment with uninfected mosquito bite vaccinations (n=5)
- Arm 5: Infectivity controls (n=6)

Eighteen vaccinated subjects (Arms 1-3) and 11 control subjects (Arms 4 and 5) underwent CHMI. Upon becoming TBS-positive, subjects were treated with atovaquone/proguanil. Twenty-six of 29 subjects became TBS positive including all control subjects. *Amongst Arm 4 and Arm 5 control subjects (n=11)*, all experienced ≥1 malaria-related AE post-CHMI, and all became biomarker positive [average 6.9 days (95%CI 6.7-7.1)] before becoming TBS positive [average 9.2 days (95%CI: 8.4-10.0 days)] for a 2.3 day acceleration by RT-PCR (95%CI: 1.5-3.1 days) (**Table S8**). The first malaria-related symptom of any grade in control participants occurred on average 8.2 days post-CHMI (95%CI: 7.2-9.1 days) and the first Grade 2 malaria-related symptom at 8.8 days (95%CI: 7.9-9.6 days). Using a modeled treatment threshold of ≥250 estimated parasites/mL, RT-PCR accelerated infection detection by 1.9 days (95%CI 1.2-2.6 days)

compared to TBS and 0.9 days (95%CI 0.2-1.6 days) compared to malaria-related symptoms.

Amongst 18 vaccinated participants, both complete and partial protection were demonstrated. Three participants in the PQ/CQ group completely protected against CHMI were excluded from further analysis since there were no positive TBS or biomarker tests for these participants. An additional subject was TBS-positive once and received treatment, but the participant was biomarker-negative and asymptomatic throughout the CHMI -- the positive TBS was later deemed a false positive and this subject was excluded from further analysis. Therefore, n=17 vaccinated subjects are included herein. The CQ ITV Arm 3 showed delayed patency in 3/3 participants (TBS positive on days 13-16). Amongst vaccinated participants that eventually became TBS positive (excluding one false positive noted above), 14 subjects became biomarker positive [average 7.5 days (95%CI 6.9-8.1 days)] and later TBS positive [average 11.4 (95%CI: 10.0-12.7 days)] for a 3.9 day acceleration by RT-PCR (95%CI: 2.9-4.8 days) (**Table S9**).

Amongst vaccinees, the first malaria-related symptom of any grade occurred on average 9.8 days post-CHMI (95%CI: 8.5-11.1 days) and the first Grade 2 malaria-related symptom at 10.0 days (95%CI: 8.2-11.8 days). Using a modeled treatment threshold of ≥250 estimated parasites/mL, RT-PCR accelerated infection detection by 2.6 days (95%CI 1.7-3.5 days) compared to TBS and 1.1 days (95%CI -0.1-2.2 days) compared to malaria-related symptoms.

NIH PfSPZ-CVac PYR study

The NIH Laboratory of Malaria Immunology and Vaccinology (LMIV) PfSPZ-Cvac PYR study (NIAID Protocol 15-I-0169) was a single center, Phase 1 study of ITV consisting of wild-type, aseptic, purified, cryopreserved *Pf* sporozoites (Sanaria® PfSPZ Challenge) administered by DVI in conjunction with CQ and pyrimethamine (PYR). The pilot and main phases are below with number of enrolled subjects completing CHMI in parentheses:

- Arm 1a (pilot phase) & Arm 2 (main phase): PYR/CQ ITV three times (n=11)
- Arm 3: CQ ITV three times (n=5)
- Arm 4: Infectivity controls (n=5)

After enrollment and ITV, 21 malaria-naïve healthy adult subjects underwent CHMI with 3200 PfSPZ by DVI (16 vaccinated and 5 infectivity controls). Two positive real time NIH Clinical Center *Plasmodium* 18S rDNA PCR results on separate days *or* one positive peripheral TBS were used for initiate treatment with atovaquone/proguanil treatment as in MC-003.

Fifteen of 21 challenged subjects met treatment criteria (TBS-positive and/or NIH PCR-positive), while six vaccinated subjects were completely protected. Of the treated group, six were treated on the basis of positive TBS. Amongst all treated subjects, 12/15

experienced ≥1 AE consistent with malaria infection (including 5/6 TBS-positive subjects).

For analysis, infected subjects were stratified as “TBS-positive” and “all treated”. *Amongst TBS-positive subjects*, 5/6 experienced malaria-related AEs post-CHMI. TBS positivity began on average at 13.5 days (95%CI: 11.0-16.0 days) with RT-PCR becoming positive earlier, on average at day 9.2 post-CHMI (95%CI: 6.1-12.2 days) for a 4.3 day acceleration by RT-PCR (95%CI: 3.1-5.6 days) (**Table S10**). In this group, the first malaria-related symptom of any grade occurred on average at 12.8 days (95%CI: 10.4-15.1 days) and the first Grade 2 malaria-related symptom at 13.8 days (95%CI: 12.2-15.3 days). RT-PCR estimated parasite densities were similar to TBS-determined densities on the day of patency. Using a modeled treatment threshold of ≥250 estimated parasites/mL, RT-PCR accelerated infection detection by 1.8 days (95%CI: 0.6-3.1 days) compared to TBS and 2.3 days (95%CI: 0.7-3.8 days) compared to symptoms. *Amongst all treated subjects*, 10/15 subjects experienced one or more malaria-related AEs post-CHMI. Fifteen subjects who were treated [average 12.0 days (95%CI: 10.8-13.2 days)] became biomarker positive on average at 8.6 days (95%CI: 7.6-9.6 days) (**Table S11**). The first malaria-related symptom of any grade occurred on average at 11.3 days post-CHMI (95%CI: 9.6-13.0 days) and the first Grade 2 malaria-related symptom on 13.5 days (95%CI: 11.9-15.1 days). At a modeled treatment threshold of ≥250 estimated parasites/mL, RT-PCR detected infections 2.7 days before symptom onset.

When protective efficacy was evaluated by vaccination arms, partial protection was best associated with the time to first positive biomarker ≥250 estimated parasites/mL although the association did not reach statistical significance because of the limited number of subjects available. The first day of positive RT-PCR and the density of the first positive result were not statistically associated with delayed TBS positivity except when TBS patency was delayed to Day 13 or later. Six subjects were completely protected and showed complete biomarker and TBS agreement. Overall, the 18S rRNA biomarker outperformed TBS for infection detection in the six TBS-positive subjects and confirmed complete protection in all TBS-negative participants.

SUPPLEMENTAL FIGURES

Figure S1. Pan-*Plasmodium* and *P. falciparum* specific primers/probes aligned to consensus sequences of asexual-type 18S rRNAs of major *Plasmodium* species

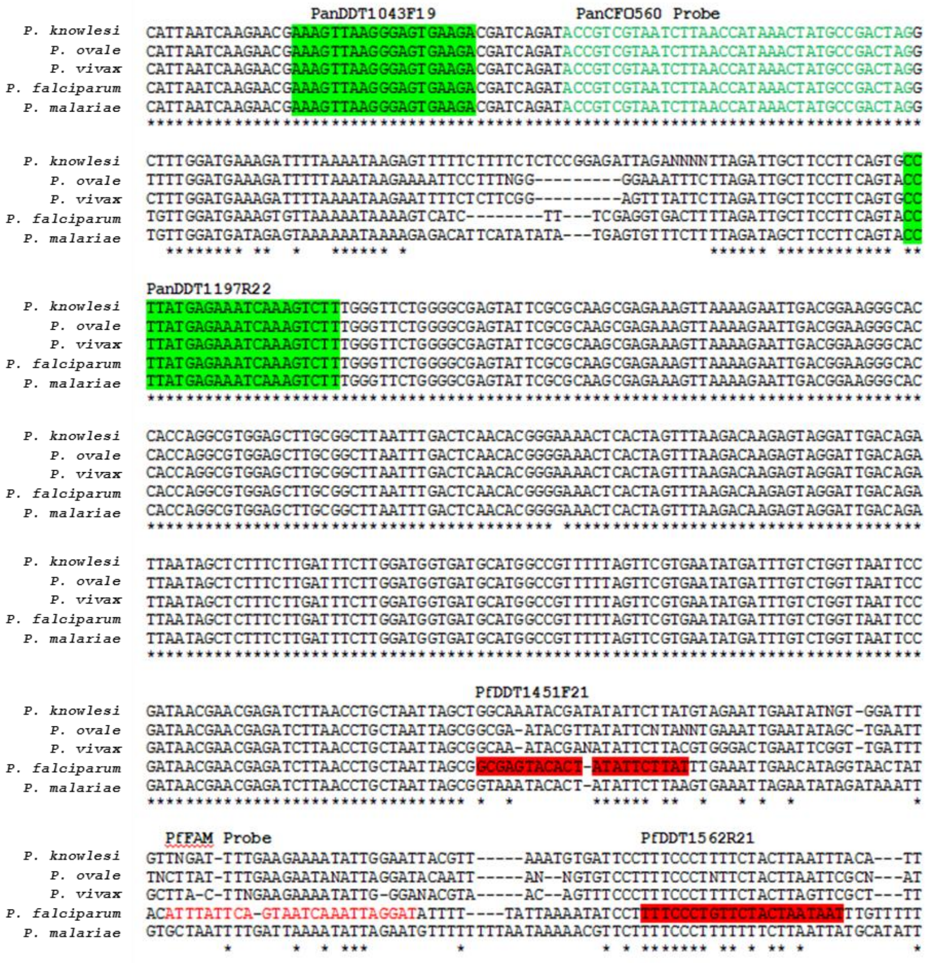

Alignment of partial reference sequences for A-type 18S rRNA genes of *P. falciparum, P. vivax, P. malariae, P. ovale* and *P. knowlesi* across the pan-*Plasmodium* and *P. falciparum*-specific amplicon region with primers and probes. Asterisks indicate 100% conserved sequences for all sequences shown. Solid green fill indicates the position of forward and reverse pan-*Plasmodium* primers and green text indicates the position of the corresponding probe (with corresponding labels). Solid red fill indicates the position of forward and reverse *P. falciparum*-specific primers and red text indicates the position of the corresponding probe (with corresponding labels).

Figure S2. Pan-*Plasmodium* and *P. falciparum* specific primers/probes alignment to *P. falciparum* sexual-and asexual-type 18S rRNAs sequences

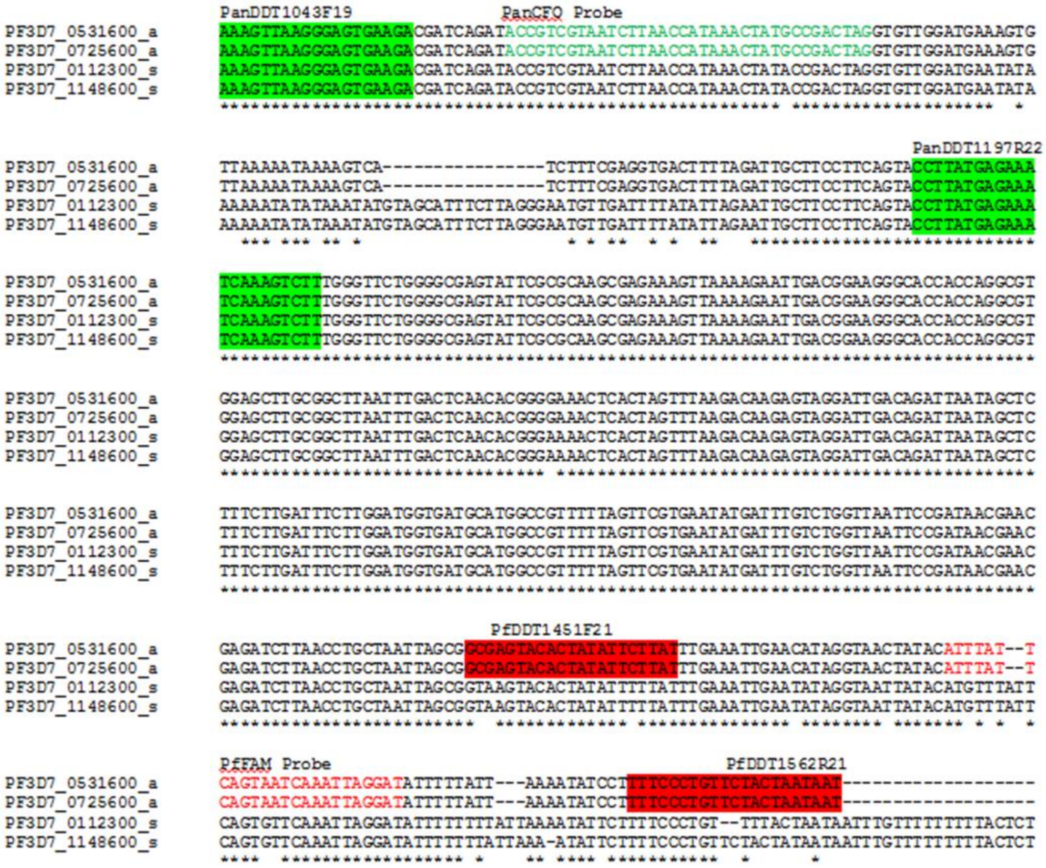

Partial reference sequences for the two A-type (PF3D7_0513600 and PF3D7_0725600) and two S-type (PF3D7_0112300 and PF3D7_1148600) 18S rRNA genes of *Pf* aligned to the pan-*Plasmodium* and *Pf*-specific primers and probes. Asterisks represent sequences conserved across all species. Solid green and red fill indicate the positions of pan-*Plasmodium* and *Pf*-specific reagents (with corresponding labels) as in Figure S1.

Figure S3. Bland-Altman difference plots for *P. falciparum* (A) and pan-*Plasmodium* (B) qRT-PCR

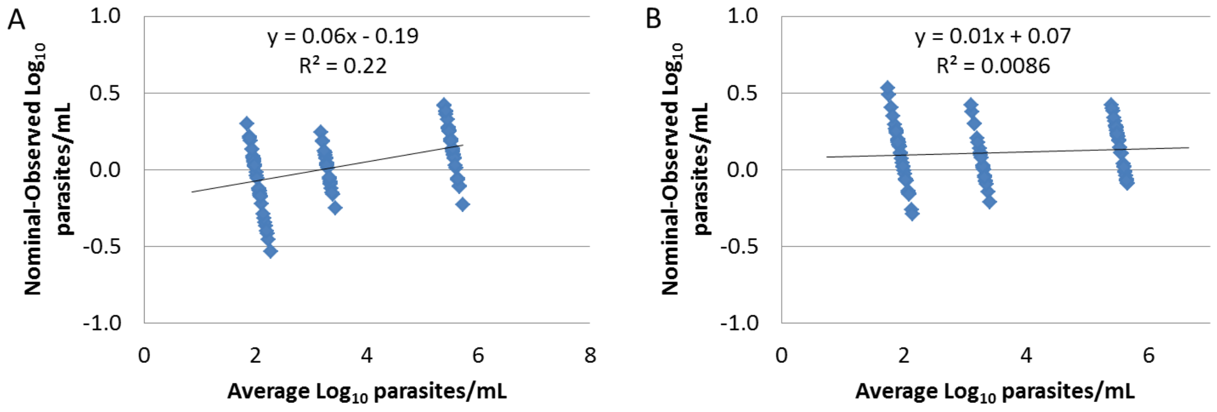

Samples ranging from $1x10^2$ parasites/mL to $4x10^5$ parasites/mL (relevant range for CHMI studies) were tested for biomarker-based differences (bias) from known (nominal) densities. qRT-PCR data are presented as log_{10} parasites/mL. This data supports the decision to quantify using the pan-*Plasmodium* channel since this channel had the least average and concentration-dependent bias across this range of parasite densities.

Figure S4. Stability of *Plasmodium* 18S rRNA in whole blood freeze-thaw experiments

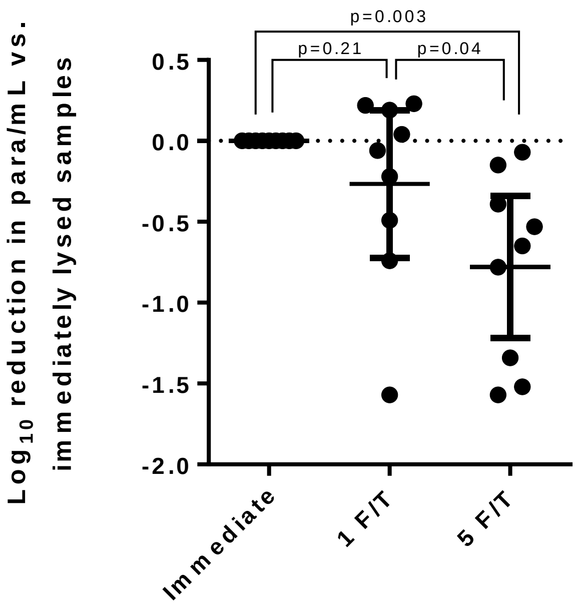

F/T, freeze-thaw

Figure S5. Biomarker assays used for discrepant analyses

A

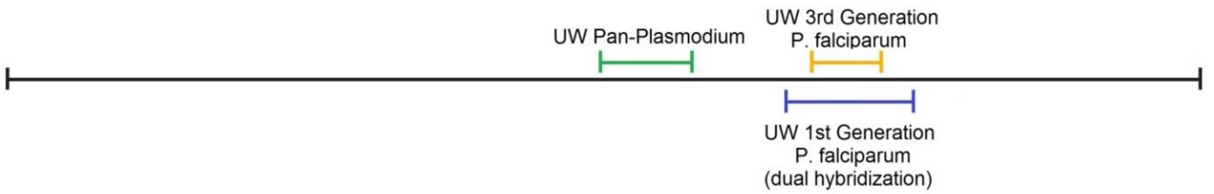

B

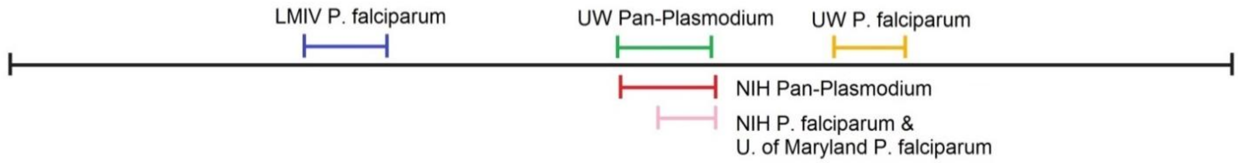

A. Assays applied to the MC-001 discrepant analysis. Graphic depicts full-length *P. falciparum* 18S rRNA (5'→3'). All primers and probes bind A-type 18S rRNA of *P. falciparum* with 100% homology. **B.** Assays applied to the NIH CVac discrepant analysis. Graphic depicts full-length *P. falciparum* 18S rRNA as in A. **'**UW 3rd generation' refers to the assay reagents described in the Methods of this manuscript. LMIV *P. falciparum* rDNA qPCR detects the S-type 18S rRNA gene (probe also matches A-type sequence with 100% homology). NIH Pan/Pf 18S rDNA PCR test and U. Maryland 18S rRNA qRT-PCR test use the same Reverse Primer. U. Maryland Pf and NIH Pf primers/probe are identical although the U. Maryland assay performs reverse transcription PCR whereas the NIH assay is PCR only.

SUPPLEMENTAL TABLES

Table S1. Data summary of blood smears and 18S rRNA/rDNA NATs in published CHMI trials

References	Immune status	CHMI Route	Subjects evaluated ?	TBS+ (%+)	Mean TBS TTP (range)	Biomarker test	Mean $\Delta TTP_{NAT-TBS}$	Mean NAT TTP (range)
32,38	Naïve	MOZ (4-7 bites)	20	20 (100%)	9.0** (7.3-10.3)	PCR	2.0	7.0**& (6.3-7.0)
35	Naïve	MOZ (5 bites)	5	5 (100%)	9.2 (7.0-11.0)	PCR	2.7	6.5 (ND)
36	Naïve	MOZ (5 bites)	5	4 (80%)	ND (7.0-12.0)	PCR	3.0	6.5 (ND)
34	Naïve	MOZ (5 bites)	6	6 (100%)	10.0 (9.0-11.0)	PCR	3.0	7.0 (7.0-7.0)
22 (VAC039)	Naïve	MOZ (5 bites)	6	6 (100%)	10.2 (9.0-11.5)	PCR	3.1	7.1 (7.0-7.5)
34	Vaccinated (AMA1 AS01B)	MOZ (5 bites)	16	16 (100%)	10.2 (9.0-11.0)	PCR	2.6	7.6 (7.0-9.0)
29	Naïve	MOZ (5 bites)	12	11 (92%)	10.4 (7.2-13.5)	PCR	2.2	8.2 (7.0-11.9)
40	Naïve	MOZ (3 bites)	19	19 (100%)	10.7 (8.8-13.9)	PCR	3.3	7.4 (6.7-11.9)
16	Naïve	DVI (3200)	4	4 (100%)	10.5 (10.0-12.0)	PCR	2.0	8.5 (7.0-9.0)
37	Vaccinated (PfSPZ ID)	MOZ (5 bites)	43	41 (95%)	10.7* (9.0-12.0)	PCR	2.8	8.0* (7.0-11.0)
23	Naïve	MOZ (3-5 bites)	12	12 (100%)	10.9 (9.0-12.0)	PCR	3.1	7.8 (ND)
30	Naïve	DVI (3200)	13	13 (100%)	11.0** (10.8-17.9)	RT-PCR	4.1	6.9** (6.0-7.0)
44	Vaccinated (PfSPZ-Cvac ID)	MOZ (5 bites)	10	8 (80%)	11.1 (10.5-15.0)	PCR	2.8	8.3 (7.0-10.5)
38	Naïve	MOZ (5 bites)	65	65 (100%)	11.2** (8.0-14.5)	PCR	4.7	7.5**& (6.5-11.0)
10,21	Naïve	MOZ (5 bites)	6	6 (100%)	11.2 (9.0-14.0)	RT-PCR	3.7	7.5 (ND)
20	Naïve	DVI (3200)	15	15 (100%)	11.2 (10.4-12.5)	PCR	2.4	8.8 (7.0-11.0)
22 (VAC039)	Vaccinated (Chad63-MVA)	MOZ (5 bites)	36	35 (97%)	11.2 (9.0-18.0)	PCR	3.7	7.5 (6.5-14.5)
22 (VAC037)	Naïve	MOZ (5 bites)	6	6 (100%)	11.3 (9.0-12.5)	PCR	3.9	7.8 (7.0-9.0)
43	Naïve	DVI (3200)	6	6 (100%)	11.5 (10.4-12.3)	PCR	3.1	8.3 (6.9-9.1)
42	Naïve	MOZ (5 bites)	36	33 (91.7%)	10.5-12.5 (8-14)	PCR	3.5	8.0 (7.0-9.0)
37	Naïve	MOZ (5 bites)	18	18 (100%)	11.5* (11.0-14.0)	PCR	2.9	8.6* (7.0-14.0)
33	Naïve	MOZ (5 bites)	5	5 (100%)	11.8 (10.5-14.5)	PCR	4.3	7.5 (ND)
45	Naïve	DVI (3200)	4	4 (100%)	11.8 (11-14)	PCR	3.8	8.0 (7-11)
41	Naïve	MOZ (5 bites)	16	16 (100%)	11.3-12.5 (0.93-1.33^)	PCR	5.2	6.7 (6.4-7.0)

39	Vaccinated (DNA/Ad CSP AMA1)	MOZ (5 bites)	15	11 (73%)	11.9 (10.0-14.0)	PCR	4.3	7.6 (7.0-9.0)
29[H]	Vaccinated (PfSPZ IV)	MOZ (5 bites)	15	3 (20%)	12.0 (10.9-12.6)	PCR	1.1	10.9 (10.0-11.9)
33	Vaccinated (FFM ME-TRAP+PEV3A)	MOZ (5 bites)	24	24 (100%)	12.4 (9.0-20.0)	PCR	4.9	7.5 (ND)
30[H]	Vaccinated (PfSPZ-Cvac IV)	DVI (3200)	27	9 (33%)	12.6** (10.9-14.6)	RT-PCR	5.6	7.0** (6.0-11.0)
39	Naïve	MOZ (5 bites)	6	6 (100%)	12.7 (11.0-16.0)	PCR	5.6	7.1 (7.0-8.0)
44	Naïve	MOZ (5 bites)	5	5 (100%)	12.9 (10.5-16.0)	PCR	5.2	7.7 (7.0-10.5)
16	Vaccinated (VRC312 PfSPZ IV at 21 weeks)	DVI (3200)	6	4 (67%)	13.5 (12.0-14.0)	PCR	2.5	11.0 (9.0-12.0)
36[H]	Vaccinated (Pf spz mosquito bites + CQ)	MOZ (5 bites)	6	2 (33%)	16.5 (15.0-18.0)	PCR	1.3	15.3 (13.5-17.0)

References refer to main manuscript references.
*Geometric mean; **Median; ^standard deviation; &Pers. comm. Meta Roestenberg and Robert Sauerwein; Hhigh efficacy designation
TBS, thick blood smear; TTP, time to positivity; ND, no data; MOZ, *P. falciparum*-infected mosquito bites; DVI, direct venous injection of PfSPZ Challenge

Table S2. Pan-*Plasmodium* assay primer/probe conservation to available GenBank sequences

Species	Sequences evaluated	*P. falciparum* 100% conservation	Pan-*Plasmodium* 100% conservation
P. falciparum	10	10/10 PfDDT1451F21<br>10/10 probe<br>10/10 PfDDT1562R21	10/10 PanDDT1043F19<br>10/10 probe<br>10/10 PanDDT1197R22
P. vivax	19	N/A	19/19 PanDDT1043F19<br>18/19 probe[a] (1/19 1bp∆)<br>18/19 PanDDT1197R22
P. ovale wallikeri	13[b]	N/A	13/13 PanDDT1043F19<br>11/13 probe<br>11/13 PanDDT1197R22
P. ovale curtisi	7	N/A	7/7 PanDDT1043F19<br>7/7 probe<br>7/7 PanDDT1197R22
P. malariae	14	N/A	14/14 PanDDT1043F19<br>14/14 probe<br>14/14 PanDDT1197R22
P. knowlesi	132[c]	N/A	131/132 PanDDT1043F19<br>129/132 probe<br>127/132 PanDDT1197R22
P. brasilianum	14	N/A	14/14 PanDDT1043F19<br>13/14 probe (1/14 1bp∆)<br>14/14 PanDDT1197R22

[a] There were 1 bp substitutions in each of the probe and reverse primer sequence that occurred in 1/19 *P. vivax* sequences. The substitutions occurred in the same sequence (DQ660817.1) and this sequence was A-type but divergent from the other 18 available *P. vivax* sequences.

[b] Two *P. ovale wallikeri* sequences align to A-type genes more than S-type genes but diverge from the 11 other *P. ovale wallikeri* and 7 *P. ovale custisi* A-type sequences and were considered outliers.

Table S3. Precision analysis

Control	Samples per run	Total runs	Log_{10} copies/mL whole blood (parasites/mL)	Pan-*Plasmodium* assay		*P. falciparum* assay	
				Intra-assay %CV within run (95% CI)	Inter-assay %CV within lab (95% CI)	Intra-assay %CV within run (95% CI)	Inter-assay %CV within lab (95% CI)
High density	2	20	9.47 ($4x10^5$)	0.79% (0.60-1.14%)	1.60% (1.31-2.06%)	1.14% (0.87-1.65%)	1.70% (1.39-2.18%)
Medium density	2	6	7.17 ($2x10^3$)	1.19% (0.77-2.63%)	2.51% (1.97-3.47%)	0.62% (0.40-1.36%)	1.83% (1.44-2.53%)
Low density	2	20	5.87 ($1x10^2$)	2.19% (1.67-3.16%)	3.36% (2.75-4.31%)	2.26% (1.73-3.26%)	3.46% (2.83-4.44%)
Acceptance criteria				*<10%*	*<15%*	*<10%*	*<15%*

Precision calculations used log_{10} copies/mL of blood values in accordance with FDA biomarker qualification guidance.

Table S4. Analytical sensitivity analysis

Clinical samples diluted in whole blood			
**Nominal parasites/mL**	**Number of replicates tested**	***P. falciparum*** **# detected (%)**	**Pan-*Plasmodium*** **# detected (%)**
250	10	10 (100%)	10 (100%)
100	10	10 (100%)	10 (100%)
50	20	19 (95%)	19 (95%)
20	20	19 (95%)	19 (95%)
10	20	14 (70%)	14 (70%)
6	21	3 (14%)	3 (14%)
**Full-length Armored RNA calibrator in whole blood**			
**Copies/mL***	**Number of replicates tested**	***P. falciparum*** **# detected (%)**	**Pan-*Plasmodium*** **# detected (%)**
$1.48x10^5$	21	21 (100%)	21 (100%)
$5.3x10^4$	20	20 (100%)	20 (100%)

*$1.48x10^5$ and $5.3x10^4$ copies of Armored RNA per mL of blood are equivalent to 20 and 7 parasites/mL of whole blood, respectively.

Table S5. Limit of quantification analysis in low copy number Armored RNA samples

Limit of quantification analysis	**1.48x10^5 copies of Armored RNA/mL blood (5.17 $\log_{10}$ copies/mL)**
**Mean ($\log_{10}$ copies/mL)**	5.13
**SD ($\log_{10}$ copies /mL)**	0.20
**Bias ($\log_{10}$ copies /mL)**	0.04
**Criterion 1: TAE* = Bias + 2(SD)**	0.44
Criterion 1 < 1.0?	*Yes*
**Criterion 2*: TAE=$\sqrt{(SD^2 + Bias^2)}$**	*0.66*
Criterion 2 < 1.0?	*Yes*

SD, standard deviation; TAE, total analytical error; *As per CLSI EP17-A2 (Ref. 49) Samples consisted of Armored RNA calibrators added to whole blood to a concentration equivalent to one parasite per 50 μL whole blood sample volume (or 20 parasites/mL of blood).

Table S6. One-sided unpaired *t*-tests of pre-lysis stability

Assay (parasite density)	Temp.	24 hr	48 hr	72 hr	96 hr
**Pan-*Plasmodium* assay ($2x10^2$ p/mL)**	4°C	0.49	0.34	0.09	0.02
	24°C	0.40	0.12	0.12	0.36
***P. falciparum* assay ($2x10^2$ p/mL)**	4°C	0.45	0.38	0.07	0.02
	24°C	0.36	0.06	0.06	0.21

n=4 samples/condition/time point vs. 0 hr samples. Calculated using log_{10} estimated parasites/mL data.

Table S7. MC-001 study results

MC-001 (n=6 volunteers)		95% Confidence Interval		Range		N
Variable	Mean	Lower Limit	Upper Limit	Min	Max	
**Days to TBS positive**	11.2	9.5	12.9	9	14	6
**Days to biomarker positive**	7.7	6.4	8.9	7	10	6
**Days to any malaria-related symptoms**	9.7	6.4	13.0	6	14	6
**Days to any Grade 2 malaria-related symptoms**	12.2	10.6	13.8	10	13	5
**Days to fever**	N/A	N/A	N/A	N/A	N/A	0
**Δ(TBS – Any Biomarker Positive) (Days)**	3.5	2.6	4.4	2	4	6
**Δ(TBS - Biomarker≥20 est. p/mL) (Days)**	3.5	2.6	4.4	2	4	6
**Δ(TBS - Biomarker≥100 est. p/mL) (Days)**	3.0	1.9	4.2	2	4	6
**Δ(TBS - Biomarker≥250 est. p/mL) (Days)**	2.2	1.7	2.6	2	3	6
**Δ(TBS - Biomarker≥500 est. p/mL) (Days)**	1.8	1.0	2.6	1	3	6
**Δ(TBS - Biomarker≥1,000 est. p/mL) (Days)**	1.3	0.8	1.9	1	2	6
**Δ(TBS - Biomarker≥10,000 est. p/mL) (Days)**	0.4	-0.7	1.5	0	2	5
**Δ(Any Symptom – Any Biomarker Positive) (Days)**	2.0	-0.8	4.8	-1	5	6
**Δ(Any Symptom - Biomarker≥250 est. p/mL) (Days)**	0.7	-1.4	2.7	-2	3	6
**Δ(TBS – Any Symptom) (Days)**	1.5	-0.7	3.7	-1	4	6

TBS, thick blood smear; N, number of volunteers; NA, not applicable

Table S8. MC-003 study results – infectivity/drug controls only (Arms 4 and 5)

MC-003 (n=11 infectivity/drug control volunteers)		95% Confidence Interval		Range		N
Variable	Mean	Lower Limit	Upper Limit	Min	Max	
Days to TBS positive	9.2	8.4	10.0	7	11	11
Days to biomarker positive	6.9	6.7	7.1	6	7	11
Days to any malaria symptoms	8.2	7.2	9.1	6	11	11
Days to any Grade 2 malaria symptoms	8.8	8.0	9.6	7	11	9
Days to fever	9.9	8.7	11.0	8	12	8
Δ(TBS – Any Biomarker Positive) (Days)	2.3	1.5	3.1	0	4	11
Δ(TBS - Biomarker≥20 est. p/mL) (Days)	2.2	1.4	3.0	0	4	11
Δ(TBS - Biomarker≥100 est. p/mL) (Days)	2.2	1.4	3.0	0	4	11
Δ(TBS - Biomarker≥250 est. p/mL) (Days)	1.9	1.2	2.6	0	4	11
Δ(TBS - Biomarker≥500 est. p/mL) (Days)	1.7	1.0	2.5	0	4	11
Δ(TBS - Biomarker≥1,000 est. p/mL) (Days)	1.4	0.4	2.3	-1	4	11
Δ(TBS - Biomarker≥10,000 est. p/mL) (Days)	0.2	-0.4	0.9	-1	2	9
Δ(Any Symptom – Any Biomarker Positive) (Days)	1.3	0.3	2.2	-1	4	11
Δ(Any Symptom - Biomarker≥250 est. p/mL) (Days)	0.9	0.2	1.6	-1	2	11
Δ(TBS – Any Symptom) (Days)	1.0	0.0	2.0	-1	4	11

TBS, thick blood smear; N, number of volunteers; NA, not applicable

Table S9. MC-003 study results – vaccinated participants only

MC-003 (n=14 vaccinated volunteers+)		95% Confidence Interval		Range		N
**Variable**	**Mean**	**Lower Limit**	**Upper Limit**	**Min**	**Max**	
**Days to TBS positive**	11.4	10.0	12.7	9	16	14
**Days to biomarker positive**	7.5	6.9	8.1	7	10	14
**Days to any malaria symptoms**	9.8	8.5	11.1	6	14	14
**Days to any Grade 2 malaria symptoms**	10.0	8.2	11.8	6	15	9
**Days to fever**	10.8	8.7	12.8	9	16	8
**∆(TBS – Any Biomarker Positive) (Days)**	3.9	2.9	4.8	2	8	14
**∆(TBS - Biomarker≥20) (Days)**	3.6	2.7	4.6	2	2	14
**∆(TBS - Biomarker≥100) (Days)**	3.1	2.1	4.2	2	2	14
**∆(TBS - Biomarker≥250) (Days)**	2.6	1.7	3.5	1	7	14
**∆(TBS - Biomarker≥500) (Days)**	2.4	1.5	3.3	1	7	14
**∆(TBS - Biomarker≥1,000) (Days)**	1.6	1.1	2.0	0	2	14
**∆(TBS - Biomarker≥10,000) (Days)**	0.1	-0.3	0.5	-1	2	12
**∆(Any Symptom – Any Biomarker Positive) (Days)**	2.3	1.2	3.4	-1	5	14
**∆(Any Symptom - Biomarker≥250) (Days)**	1.1	-0.1	2.2	-3	4	14
**∆(TBS – Any Symptom) (Days)**	1.6	0.2	3.0	0	6	14

TBS, thick blood smear; N, number of volunteers; NA, not applicable
+ excludes volunteer MC-003-012 with false positive TBS and three completely protected persons

Table S10. NIH PfSPZ-Cvac PYR study results - TBS positive volunteers only

NIH PfSPZ-Cvac PYR - TBS positive only (n=6 volunteers)		95% Confidence Interval		Range		N
**Variable**	**Mean**	**Lower Limit**	**Upper Limit**	**Min**	**Max**	
**Days to TBS Positivity**	13.5	11.0	16.0	12	18	6
**Days to RTPCR Positivity**	9.2	6.1	12.2	7	15	6
**Days to Any Malaria-Related Symptoms**	12.8	10.4	15.1	11	14	4
**Days to Any Grade 2 Malaria-Related Symptoms**	13.8	12.2	15.3	13	15	4
**Days to Fever**	13.0	10.5	15.5	12	14	3
**Δ(TBS – Any Biomarker Positive) (Days)**	4.3	3.1	5.6	3	6	6
**Δ(TBS - Biomarker≥20 est. p/mL) (Days)**	4.2	2.9	5.4	3	6	6
**Δ(TBS - Biomarker≥100 est. p/mL) (Days)**	2.7	1.0	4.4	1	5	6
**Δ(TBS - Biomarker≥250 est. p/mL) (Days)**	1.8	0.6	3.1	1	4	6
**Δ(TBS - Biomarker≥500 est. p/mL) (Days)**	1.3	0.8	1.9	1	2	6
**Δ(TBS - Biomarker≥1,000 est. p/mL) (Days)**	1.3	0.8	1.9	1	2	6
**Δ(TBS - Biomarker≥10,000 est. p/mL) (Days)**	0.0	-0.9	0.9	-1	1	5
**Δ(Any Symptom – Any Biomarker Positives) (Days)**	4.8	3.2	6.3	4	6	4
**Δ(Any Symptom - Biomarker≥250 est. p/mL) (Days)**	2.3	0.7	3.8	1	3	4
**Δ(TBS – Any Symptom) (Days)**	0.0	-2.3	2.3	-2	1	4

TBS, thick blood smear; N, number of volunteers; NA, not applicable

Table S11. NIH PfSPZ-Cvac PYR study results – all treated volunteers

NIH PfSPZ-Cvac PYR all treated volunteers (n=15)		95% Confidence Interval		Range		N
**Variable**	**Mean**	**Lower Limit**	**Upper Limit**	**Min**	**Max**	
**Days to Protocol-defined treatment definition***	12.0	10.8	13.2	8	18	15
**Days to UW Biomarker Positivity**	8.6	7.6	9.6	7	15	15
**Days to Any Malaria-Related Symptoms**	11.3	9.6	13.0	7	14	10
**Days to Any Grade 2 Malaria-Related Symptoms**	13.5	11.9	15.1	12	14	5
**Days to Fever**	13.3	11.7	14.8	12	14	4
**∆(Diagnosis* – Any Biomarker Positive**) (Days)**	3.4	2.7	4.1	1.0	6.0	15
**∆(Diagnosis* - Biomarker≥20 est. p/mL) (Days)**	3.3	2.6	3.9	1.0	6.0	15
**∆(Diagnosis* - Biomarker≥100 est. p/mL) (Days)**	1.9	1.1	2.6	0.0	4.0	14
**∆(Diagnosis* - Biomarker≥250 est. p/mL) (Days)**	1.2	0.7	1.8	0.0	4.0	14
**∆(Diagnosis* - Biomarker≥500 est. p/mL) (Days)**	1.0	0.7	1.3	0.0	2.0	13
**∆(Diagnosis* - Biomarker≥1,000 est. p/mL) (Days)**	0.8	0.5	1.2	0.0	2.0	12
**∆(Diagnosis* - Biomarker≥10,000 est. p/mL) (Days)**	0.2	-0.2	0.6	0.0	1.0	5

TBS, thick blood smear; N, number of volunteers; NA, not applicable
*Definition of infection described in the study protocol.

Table S12. Comparison of TBS and biomarker-defined parasite density estimates

Variable	Statistics	MC-001 (N=6)	MC-003 Infectivity & drug controls (N=11)	MC-003 Vaccinated (N=14)	PfSPZ-Cvac PYR (N=6)	All Studies (N=37)
**Density (est. p/mL) at first positive biomarker RTPCR**	N	6	11	14	6	37
	Mean	172	2,347	559	71	950
	Min-Max	31-379	10*-10,904	10*-4,289	19-139	19-10,904
**Density (est. p/mL) at TBS positive (by TBS)**	N	6	11	14	6	37
	Mean	19,983	16,372	19,571	25,616	19,668
	Min-Max	2,330-53,700	1,700-72,000	4,000-82,000	6,000-74,000	1,700-82,000
**Density (est. p/mL) at TBS positive (by RTPCR)**	N	6	11	14	6	37
	Mean	42,001	39,812	43,102	43,308	41,979
	Min-Max	7,450-114,422	3,852-156,682	1,687-120,591	1,761-162,152	1,687-162,152

* Denotes low positive result (input at 10 est. p/mL)

Table S13. Agreement analysis for UW pan-*Plasmodium* 18S rRNA qRT-PCR compared to reference blood smears and non-reference molecular comparator assays

**MC-001 study**						
**Comparator Method**	**PPA**	**PPA 95%CI**	**NPA**	**NPA 95%CI**	**OPA**	**OPA 95%CI**
**Blood smear (Reference, n=43)**	100%	61.0-100%	45.9%	31.0-61.6%	53.5%	38.9-67.5%
**UW 1st gen *Pf* 18S rRNA (n=43)**	92.9%	77.4-98.0%	100%	79.6-100%	95.3%	84.5-98.7%
**UW 3rd gen *Pf* 18S rRNA (n=43)**	96.3%	81.7-99.3%	100%	80.6-100%	97.7%	87.9-99.6%
**NIH PfSPZ-Cvac PYR study**						
**Comparator Method**	**PPA**	**PPA 95%CI**	**NPA**	**NPA 95%CI**	**OPA**	**OPA 95%CI**
**Blood smear (Reference, n=177)**	100%	61.0-100%	69.6%	81.3-91.3%	87.6%	81.9-91.6%
**LMIV Pf 18S rDNA (n=187)**	100%	78.5-100%	82.8%	86.9-95.1%	92.5%	87.8-95.5%
**NIH Pf 18S rDNA (n=187)**	100%	78.5-100%	86.4%	86.9-95.1%	92.5%	87.8-95.5%
**U. Maryland *Pf* 18S rRNA (n=187)**	88.3%	71.0-96.0%	96.1%	92.9-98.7%	95.7%	91.8-97.8%
**UW 3rd gen *Pf* 18S rRNA (n=187)**	95.2%	78.7-98.2%	100%	97.6-100%	98.9%	96.2-99.7%

Data is from day of CHMI through day of blood smear positivity. n = indicates the number of samples pairs for the primary assay (pan-*Plasmodium* 18S rRNA qRT-PCR) with the listed comparator assay for samples from the day of CHMI to the day of blood smear positivity. Reported limits of detection: thick blood smears 5,000-20,000 parasites/mL (Garcia LS. Malaria. 2010. *Clin Lab Med*. 30(1):93-129), UW 1st generation assay 20 parasites/mL (Ref. 10), LMIV 20 parasites/mL (J. Neal, pers. comm.), NIH 500 parasites/mL (Ref. 29), U. Maryland 40 parasites/mL (Ref. 28), 3rd generation assays 20 parasites/mL (this manuscript). PPA, positive percent agreement; NPA, negative percent agreement; OPA, overall percent agreement.
